# Molecular, Physiological, and Motor Performance Defects in DMSXL Mice Carrying >1,000 CTG Repeats from the Human DM1 Locus

**DOI:** 10.1371/journal.pgen.1003043

**Published:** 2012-11-29

**Authors:** Aline Huguet, Fadia Medja, Annie Nicole, Alban Vignaud, Céline Guiraud-Dogan, Arnaud Ferry, Valérie Decostre, Jean-Yves Hogrel, Friedrich Metzger, Andreas Hoeflich, Martin Baraibar, Mário Gomes-Pereira, Jack Puymirat, Guillaume Bassez, Denis Furling, Arnold Munnich, Geneviève Gourdon

**Affiliations:** 1 Inserm U781, Université Paris Descartes-Sorbonne Paris Cité, Institut Imagine, Hôpital Necker-Enfants Malades, Paris, France; 2 Institut de Myologie, Université Paris 6 UMR S974, Inserm U974, CNRS UMR 7215, GH Pitié-Salpêtrière, Paris, France; 3 Généthon, Evry, France; 4 Inserm U955, Département de Neurosciences, Faculté de Médecine, Université Paris XII, Créteil, France; 5 Université Paris Descartes-Sorbonne Paris Cité, Paris, France; 6 F. Hoffmann-La Roche, CNS Pharma Research and Development, Basel, Switzerland; 7 Leibniz-Institute for Farm Animal Biology (FBN), Dummerstorf, Germany; 8 UPMC Univ Paris 06, UM 76, Institut de Myologie and Inserm, U974 and CNRS, UMR7215, Paris, France; 9 Human Genetics Research Unit, Laval University, Québec City, Québec, Canada; The Hospital for Sick Children and University of Toronto, Canada

## Abstract

Myotonic dystrophy type 1 (DM1) is caused by an unstable CTG repeat expansion in the 3′UTR of the DM protein kinase (*DMPK*) gene. *DMPK* transcripts carrying CUG expansions form nuclear foci and affect splicing regulation of various RNA transcripts. Furthermore, bidirectional transcription over the *DMPK* gene and non-conventional RNA translation of repeated transcripts have been described in DM1. It is clear now that this disease may involve multiple pathogenic pathways including changes in gene expression, RNA stability and splicing regulation, protein translation, and micro–RNA metabolism. We previously generated transgenic mice with 45-kb of the DM1 locus and >300 CTG repeats (DM300 mice). After successive breeding and a high level of CTG repeat instability, we obtained transgenic mice carrying >1,000 CTG (DMSXL mice). Here we described for the first time the expression pattern of the *DMPK* sense transcripts in DMSXL and human tissues. Interestingly, we also demonstrate that *DMPK* antisense transcripts are expressed in various DMSXL and human tissues, and that both sense and antisense transcripts accumulate in independent nuclear foci that do not co-localize together. Molecular features of DM1-associated RNA toxicity in DMSXL mice (such as foci accumulation and mild missplicing), were associated with high mortality, growth retardation, and muscle defects (abnormal histopathology, reduced muscle strength, and lower motor performances). We have found that lower levels of IGFBP-3 may contribute to DMSXL growth retardation, while increased proteasome activity may affect muscle function. These data demonstrate that the human DM1 locus carrying very large expansions induced a variety of molecular and physiological defects in transgenic mice, reflecting DM1 to a certain extent. As a result, DMSXL mice provide an animal tool to decipher various aspects of the disease mechanisms. In addition, these mice can be used to test the preclinical impact of systemic therapeutic strategies on molecular and physiological phenotypes.

## Author Summary

Myotonic dystrophy type 1 (DM1) is caused by the abnormal expansion of a CTG repeat located in the DM protein kinase (*DMPK*) gene. *DMPK* transcripts carrying CUG expansions form toxic nuclear foci that affect other RNAs. DM1 involve multiple pathogenic pathways including changes in gene expression, RNA stability and splicing regulation, protein translation, and micro–RNA metabolism. We previously generated transgenic mice carrying the human DM1 locus and very large expansions >1,000 CTG (DMSXL mice). Here we described for the first time, the expression pattern of the *DMPK* sense transcripts in DMSXL and human tissues. We also demonstrate that *DMPK* antisense transcripts are expressed in various tissues from DMSXL mice and human. Both sense and antisense transcripts form nuclear foci. DMSXL mice showed molecular DM1 features such as foci and mild splicing defects as well as muscles defects, reduced muscle strength, and lower motor performances. These mice recapitulate some molecular features of DM1 leading to physiological abnormalities. DMSXL are not only a tool to decipher various mechanisms involved in DM1 but also to test the preclinical impact of systemic therapeutic strategies.

## Introduction

Myotonic dystrophy type I (DM1) is a dominantly inherited disorder, highly variable and associated with multisystemic symptoms. The adult onset form of the condition typically presents distal muscle weakness, myotonia, cardio-respiratory problems, presenile cataracts, hypersomnia, hyperinsulinism, testicular atrophy and early frontal balding in males [1]. Behavioral changes and cognitive dysfunction are also frequent. The more severe congenital form of DM1 (CDM) is characterized by general hypotonia at birth, respiratory distress, difficulties in sucking and swallowing and facial weakness. Mortality in CDM during the neonatal period has been estimated between 30% and 40% of patients [2].

The genetic mutation causing DM1 is the expansion of an unstable CTG repeat in the 3′ untranslated region (3′UTR) of a gene encoding a protein kinase (DMPK) [3]–[5]. The normal *DMPK* gene contains 5–37 CTG repeats in the 3′UTR, while all DM1 patients have repeats expanding from 50 to several thousand CTG trinucleotide repeats in CDM. The size of the CTG repeat generally increases from generation to generation in DM1 families [1].

The development of different animal models revealed that DM1 is not a simple dosage, gain-of- or loss-of-function disorder [6]. Instead, entirely novel pathological pathways at the DNA, RNA, and/or protein level may play a role in the disease manifestation [7]. The repeat expansion affects not only the expression of *DMPK*, but also the neighboring *SIX5* gene, as well as others gene products: abnormal retention of mutant *DMPK* transcripts forming foci in cell nuclei alters the metabolism of other RNAs by disturbing the function of CUG-binding proteins involved in splicing or transcription regulation [8]–[13]. In DM1 patients, a growing number of splicing defects has been identified in various tissues [14]. Clinically similar to DM1, myotonic dystrophy type 2 (DM2) is caused by the expansion of an intronic CCTG repeat in an unrelated gene. The identification of the genetic defect underlying DM2 emphasized the major role of RNA toxicity in myotonic dystrophy pathogenesis, while it raised questions about the contribution of *DMPK* and *SIX5* haploinsufficiency [15]. An antisense transcript emanating from the adjacent *SIX5* regulatory region downstream of the CTG repeat has been described [16]. This transcript is located in the CTG repeat region surrounded by CTCF binding sites. In CDM cells, the expanded allele is associated with loss of CTCF binding, propagation of heterochromatin resulting in the decrease of *SIX5* expression [16]. However, the relationship between antisense RNA, expression levels of *DMPK* and *SIX5* and CTG repeat length during development is still unclear. More recently, Zu *et al.* demonstrated that CAG expansion containing RNA can express homopolymeric polyglutamine, polyalanine, and polyserine proteins in the absence of an ATG start codon, resulting at least in the accumulation of polyglutamine expansion proteins in DM1 mouse models and human tissues [17]. The contribution of these Repeat Associated Non-ATG (RAN) translated proteins to disease pathology is unknown but it adds another level of complexity to the possible mechanisms involved in DM1.

We have previously generated transgenic mice carrying a 45-kb human genomic fragment with the *DMPK* gene and a normal 20 CTG repeat (DM20) or an expansion of 320 CTG (DM300) [18]. Analysis of the CTG repeat length in mice carrying expansions showed that the CTG repeat instability is similar to that observed in DM1 patients (strongly biased towards expansions, length- and age -dependent somatic instability), except that very large expansions are rare upon germinal transmissions [18]–[20]. Homozygous DM300 mice expressing enough toxic mutant RNA displayed myotonia, muscle histological abnormalities at old age with progressive skeletal muscle weakness associated with atrophic processes [21], [22]. However, these DM1-like symptoms were mild, variable and usually appeared in older mice. No major splicing defects were detected, limiting the use of this mouse model in the characterization of pathological processes or in therapeutic experiments. Due to the high levels of intergenerational instability in the DM300 lines, we obtained mice carrying over 1000 CTG (renamed “DMSXL”) after consecutive breeding [23].

Here, we characterized in detail the molecular and physiological features of homozygous DMSXL mice (carrying between 1000–1600 CTG repeats). We demonstrated for the first time that not only *DMPK* but also the antisense *DMPK* RNA are expressed in various tissues. Expression of mutant transcripts carrying very large expansions in DMSXL mice induced the formation of numerous RNA foci, mild splicing defects and affected muscle and motor performance. Therefore, this model is suitable not only to test systemic therapeutic strategies but also to study the impact of RNA toxicity in various tissues.

## Results

### The *DMPK* transgene carrying >1,000 CUG is expressed in various DMSXL tissues

**Figure 1:**
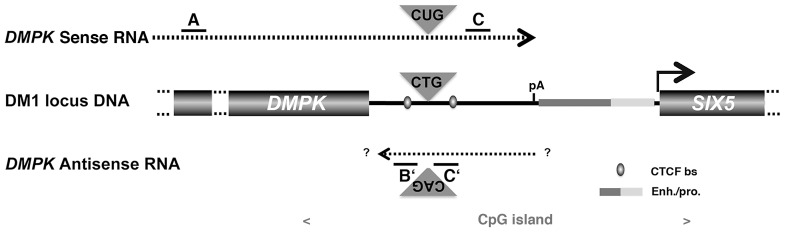
Schematic representation of the *DMPK/SIX5* region. Dashed lines represent sense and antisense *DMPK* transcript. A, B′, C and C′ represent the localization of amplicons used for qRT-PCR. CTCF bs: CTCF binding site; Enh/pro enhancer and promoter region. pA: polyadenylation site for the *DMPK* sense mRNA The *SIX5* start site is indicated with an arrow. The location of the CpG island is indicated in grey.

**Figure 2:**
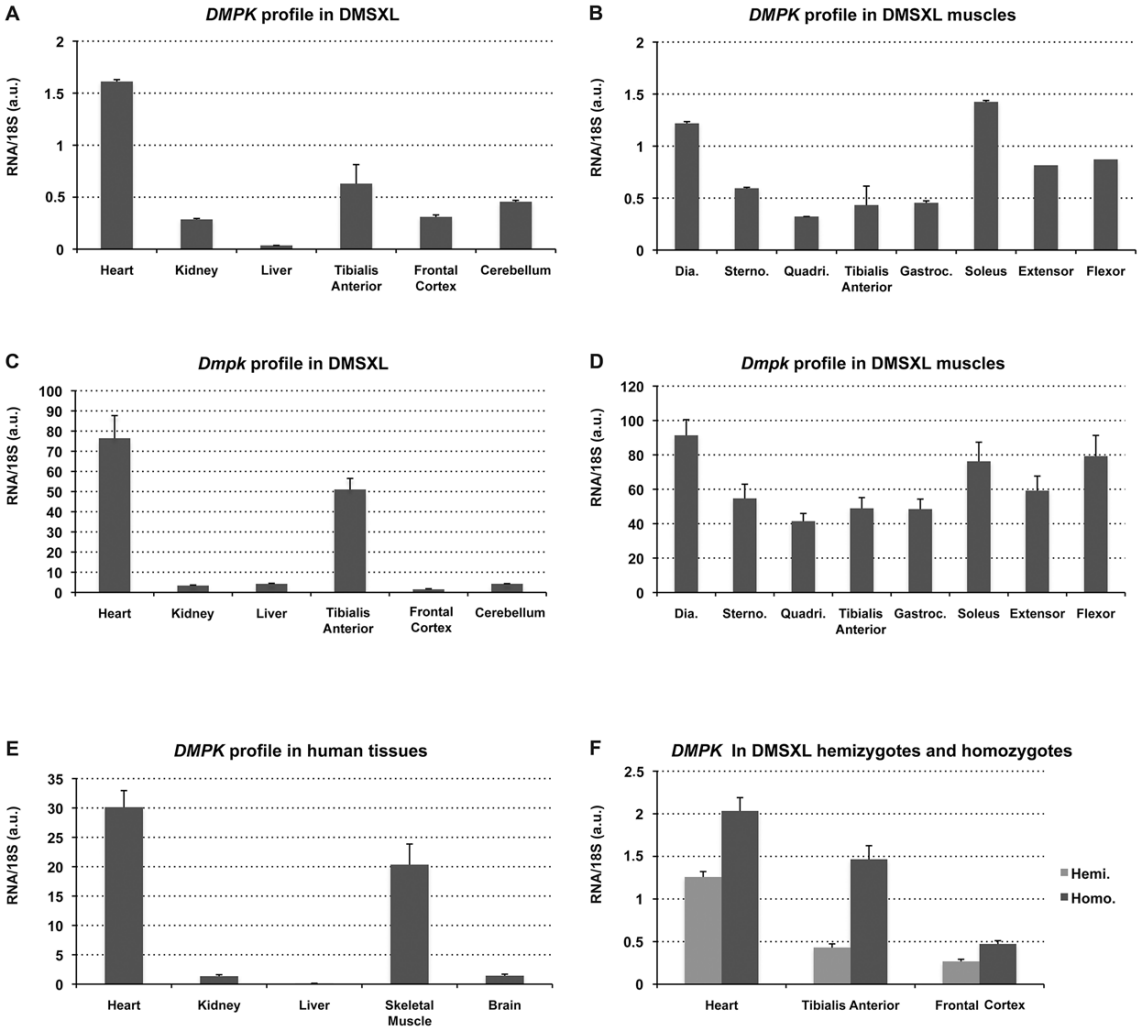
*DMPK* expression profiles. Expression of the human *DMPK* transgene was studied in various hemizygous DMSXL tissues (A) and muscles (B), in parallel with the endogenous *Dmpk* mouse gene (C and D) (n = 3). (E) Expression of *DMPK* in human tissues. Dia., Diaphragm; Sterno., Sternomastoid; Quadri, Quadriceps; TA, Tibialis Anterior; Gastroc. Gastrocnemius. (a.u.): arbitrary units. (F) Expression of *DMPK* in hemizygous (Hemi.) and homozygous (Homo.) DMSXL tissues. Data are presented as means ± standard deviation.

The expression profile of the human *DMPK* transgene in 4-month-old female DMSXL mouse tissues were determined by qRT-PCR using primers spanning exon 4 and 5 (amplicon A, Figure 1). This profile was compared to the profiles of the endogenous *Dmpk* gene in the same tissues and the *DMPK* gene in a panel of human tissues (Figure 2). The *DMPK* transgene is expressed under its own promoter. *DMPK* RNA levels in DMSXL tissues were higher in heart and in skeletal muscle, similar to endogenous *Dmpk* RNA and to *DMPK* RNA in human tissues (Figure 2A, 2C, 2E). However, the relative expression of *DMPK* between tissues was higher in frontal cortex, cerebellum and kidney in DMSXL mice. *DMPK* expression profiles were similar between hemizygous and homozygous DMSXL, with higher levels in homozygous mice as expected (Figure 2F). By qRT-PCR using hexamer RT primers and standard curves established with a known number of plasmid molecules carrying the amplicons, we estimated that endogenous *Dmpk* RNA level was about 3 times higher than the transgene *DMPK* RNA levels in DMSXL homozygous heart (Figure S1). This is in agreement with previous results obtained in DM300 mice using ribonuclease protection assay (Figure S1, [24]). On the contrary, transgene *DMPK* RNA levels were about 3 times higher than endogenous murine *Dmpk* RNA in homozygous DMSXL frontal cortex, in line with a higher relative expression of the transgene in this brain region (Figure S1). Interestingly, expression of the human *DMPK* transgene and murine *Dmpk* genes varied between muscles with higher expression in the diaphragm, sternomastoid, soleus, foreleg extensor and flexor (Figure 2B, 2D).

### The *DMPK* antisense RNA is expressed in various human and DMSXL mouse tissues

**Figure 3:**
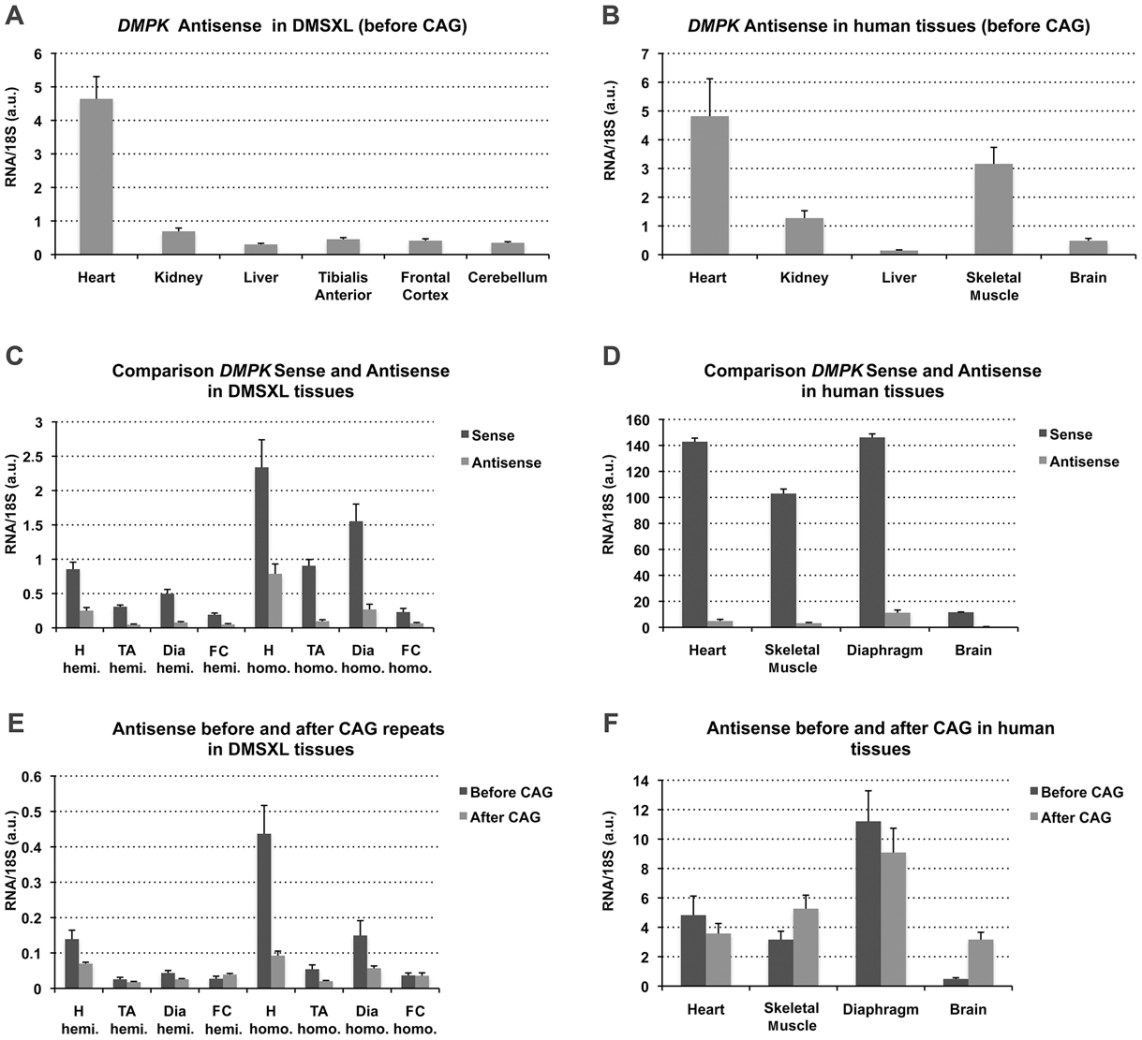
Expression of sense and antisense *DMPK* transcripts. (A–B) *DMPK* antisense expression profile in 4-month-old DMSXL homozygotes (n = 3) and human control adult tissues (commercial panel) using amplicon B′ located upstream the CAG repeat. (C–D) Comparison of *DMPK* sense and antisense transcript levels in 4-month-old DMSXL homozygotes (n = 3) and human control tissues. (E–F) Comparison of antisense transcript levels measured in 5′ (before) and in 3′ (after) of the CAG repeat using amplicons B′ and C′ in DMSXL and control human tissues. H, heart; TA, tibialis anterior; Dia, diaphragm; FC, Frontal Cortex; Hemi., Hemizygous; Homo. Homozygous. Data are presented as means ± standard deviation in arbitrary units (a.u.).

Using specific primers with linkers as described by Cho et *al* [16], we specifically reverse transcribed the *DMPK* antisense in amplicon C′ located in 5′ of the CAG repeat (Figure 1). qPCR was performed with the linker and specific complementary antisense reverse primers. The expression profile determined in homozygous DMSXL mouse tissues showed that *DMPK* antisense was expressed in various tissues with the highest expression in heart (Figure 3A). *DMPK* antisense RNA was also detected in a commercial panel of normal human tissues with the highest levels in heart and skeletal muscle (muscle type not specified) (Figure 3B). Using specific qRT-PCR and standard curves established with a known number of plasmid molecules coding the amplicons, we detected approximately 9, 3 and 3.5 fold more sense RNA than antisense RNA in homozygous DMSXL tibialis anterior, heart and frontal cortex respectively (Figure 3C). In human control tissues we estimated 33, 30 and 24 fold more sense RNA than antisense RNA in normal human muscle, heart and brain (Figure 3D). We then examined if *DMPK* antisense RNA could be detected in 3′ of the CAG repeat using specific primers in amplicon B′ (Figure 1). In DMSXL homozygotes we detected more antisense before the repeat, while in control human tissues the levels of sense and antisense RNA were similar except in brain (Figure 3E, 3F).

### Numerous nuclear foci of mutant *DMPK* transcripts are observed in various DMSXL tissues

**Figure 4:**
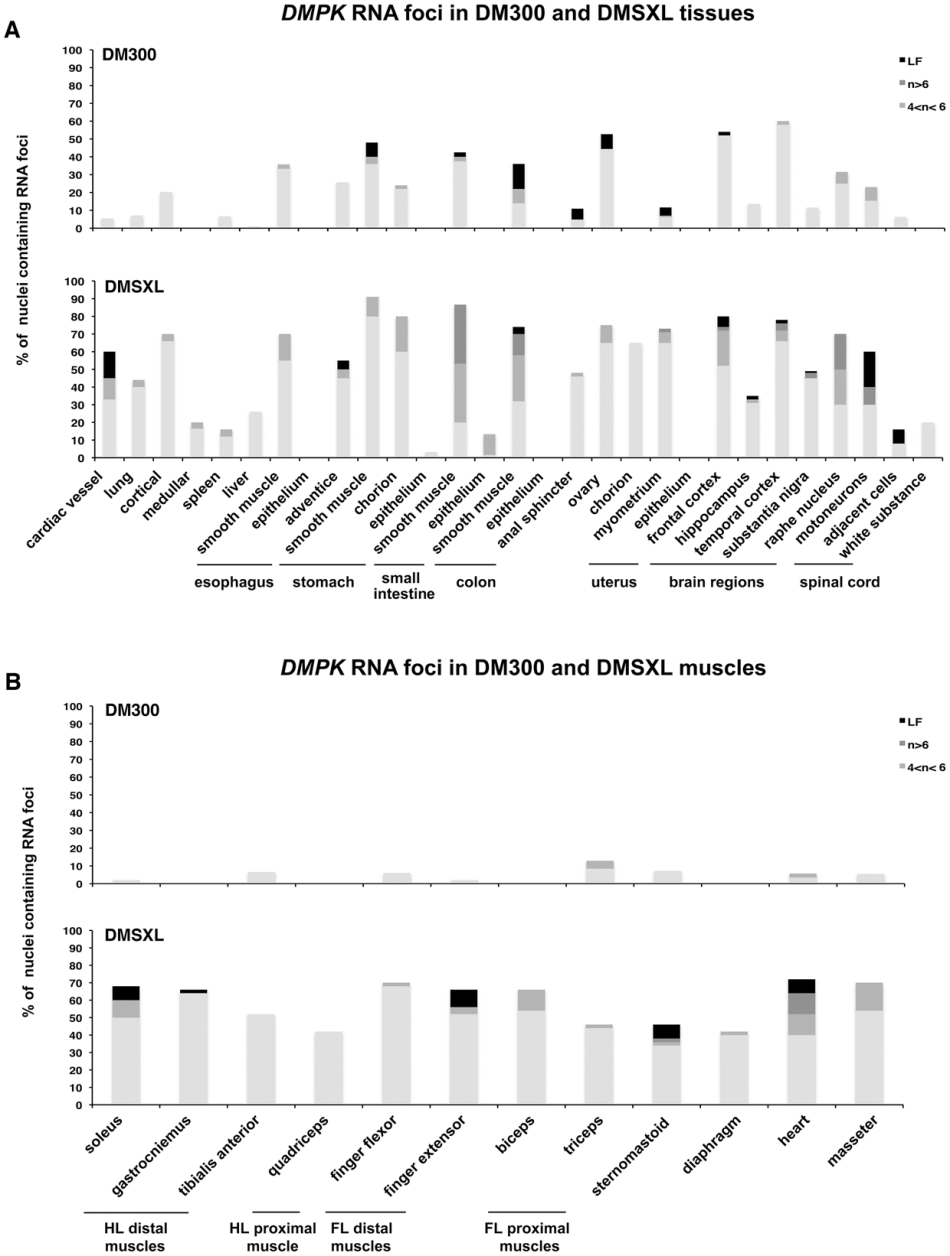
Foci distribution in mice carrying CTG expansions. The distribution and quantification of *DMPK* RNA foci in various tissues (A) and muscles (B) were compared in 8 month-old homozygous mice with 500/600 CTG (DM300) or 1000/1400 CTG (DMSXL). HL, hind leg; FL foreleg. n is for the number of foci per nucleus. LF: large foci.

We previously observed mutant *DMPK* RNA foci in various tissues from DM300 mice carrying between 400 and 600 CTG repeats [24], [25]. We then extended our analysis in a large panel of tissues from DM300 and DMSXL mice. Due to the ubiquitous expression of the human *DMPK* transgene in mice, we observed nuclear foci in the majority of the tissues tested, except in epithelia (Figure 4). Compared to the DM300 mouse tissues, DMSXL tissues showed higher levels of nuclei containing foci, ranging from 1 up to more than 6 foci per nucleus, occasionally forming very large foci. Tissues known to be affected in DM1, such as heart, skeletal muscles (including diaphragm and masseter) and brain, showed numerous foci, as well as other tissues that might be involved in disease, such as smooth muscle from the gastrointestinal tract, uterus and spinal chord (Figure 4). We also observed that the percentage of nuclei containing foci was higher in heart tissue from a one-month-old DMSXL homozygote compared with a sex-matched hemizygote from the same litter (respectively 71% versus 47%, p<0.0001, Mann-Whitney test). In DMSXL skeletal muscles and heart, we observed that sense *DMPK* mutant transcripts co-localized with foci formed by sequestrated MBNL1 and MBNL2 (Figures S2 and S3).

### Antisense *DMPK* transcripts form nuclear foci in DMSXL muscle and heart

**Figure 5:**
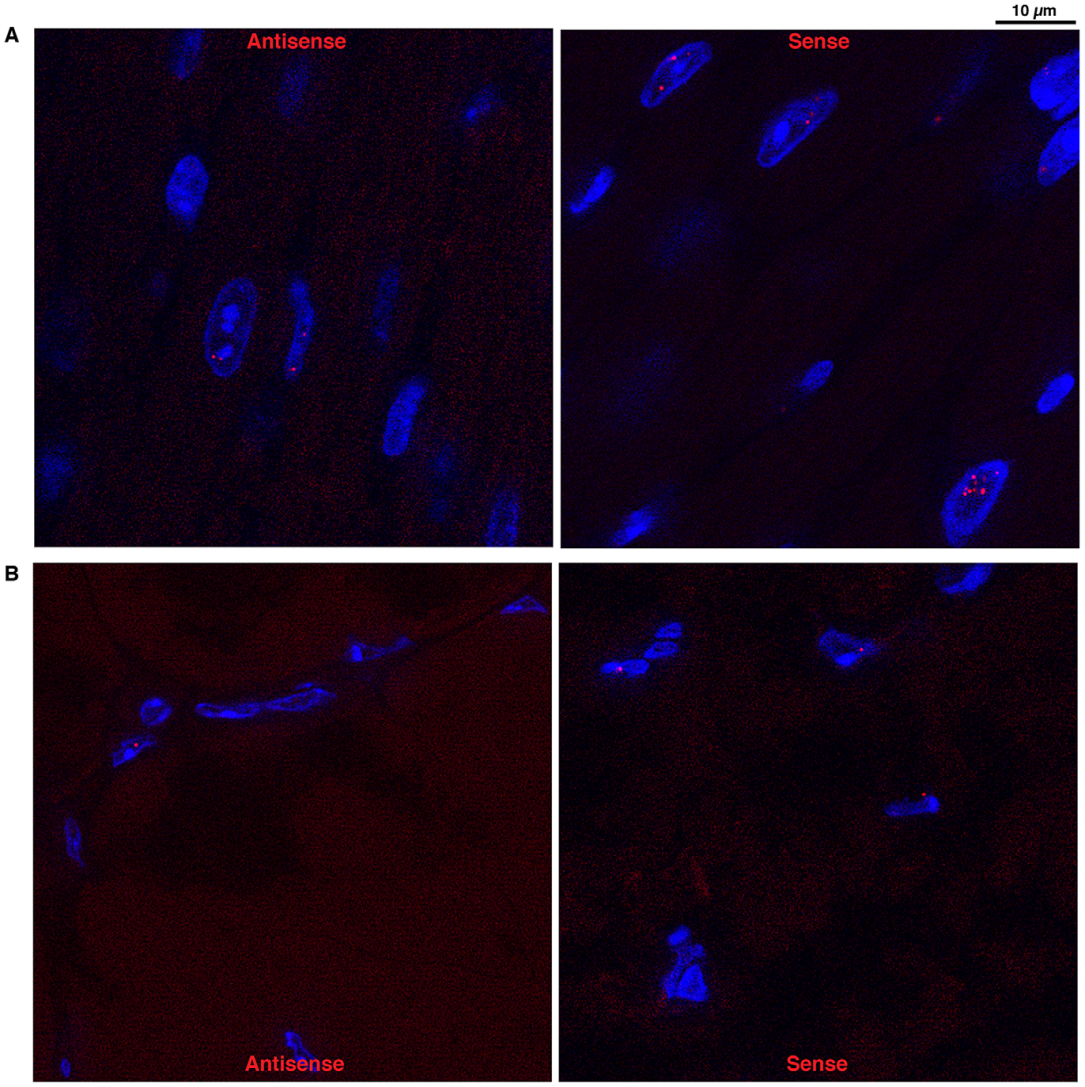
Foci accumulation was detected for both sense and antisense *DMPK* transcripts. FISH experiments using 5′-Cy3- labeled (CAG)5 or 5′-Cy3- labeled (CTG)5 probes detected respectively, in red, sense and antisense RNA foci in heart (A) and muscle (B) from DMSXL homozygote.

Using a (CTG)5 fluorescent probe we investigated if antisense RNA carrying CAG expansions could form ribonuclear inclusions in DMSXL heart and skeletal muscles. Indeed, we observed nuclear foci in both heart and gactrocnemius (Figure 5). Usually one antisense foci, and sometimes up to three foci, were detected per nucleus. However, the number of antisense foci and the percentage of nuclei showing antisense foci were lower than that observed for the sense foci (for example 17% and 8% nuclei showed antisense foci in homozygous and hemizygous one-month-old DMSXL heart respectively, versus 71% and 47% for the sense foci). Antisense foci were also observed at low frequency in human DM1 heart but not in DM20 tissues or in DMSXL after ribonuclease treatment. Antisense foci did not co-localize with sense transcripts (Figure S2A and S3A). CAG ribonuclear foci have been shown to co-localize with MBNL1 in transfected cells and transgenic mice [26], . However, using antibodies specific for MBNL1 and MBNL2 we could not detect clear co-localization and sequestration of these proteins by the CAG transcripts in DMSXL skeletal muscles and heart, only faint protein foci were sometimes observed (Figures S2B and S3B). However, in human DM1 heart samples we observed MBNL1 foci that co-localized with CAG antisense transcripts (Figure S4).

### Mild splicing defects are observed in muscle and heart from DMSXL

**Figure 6:**
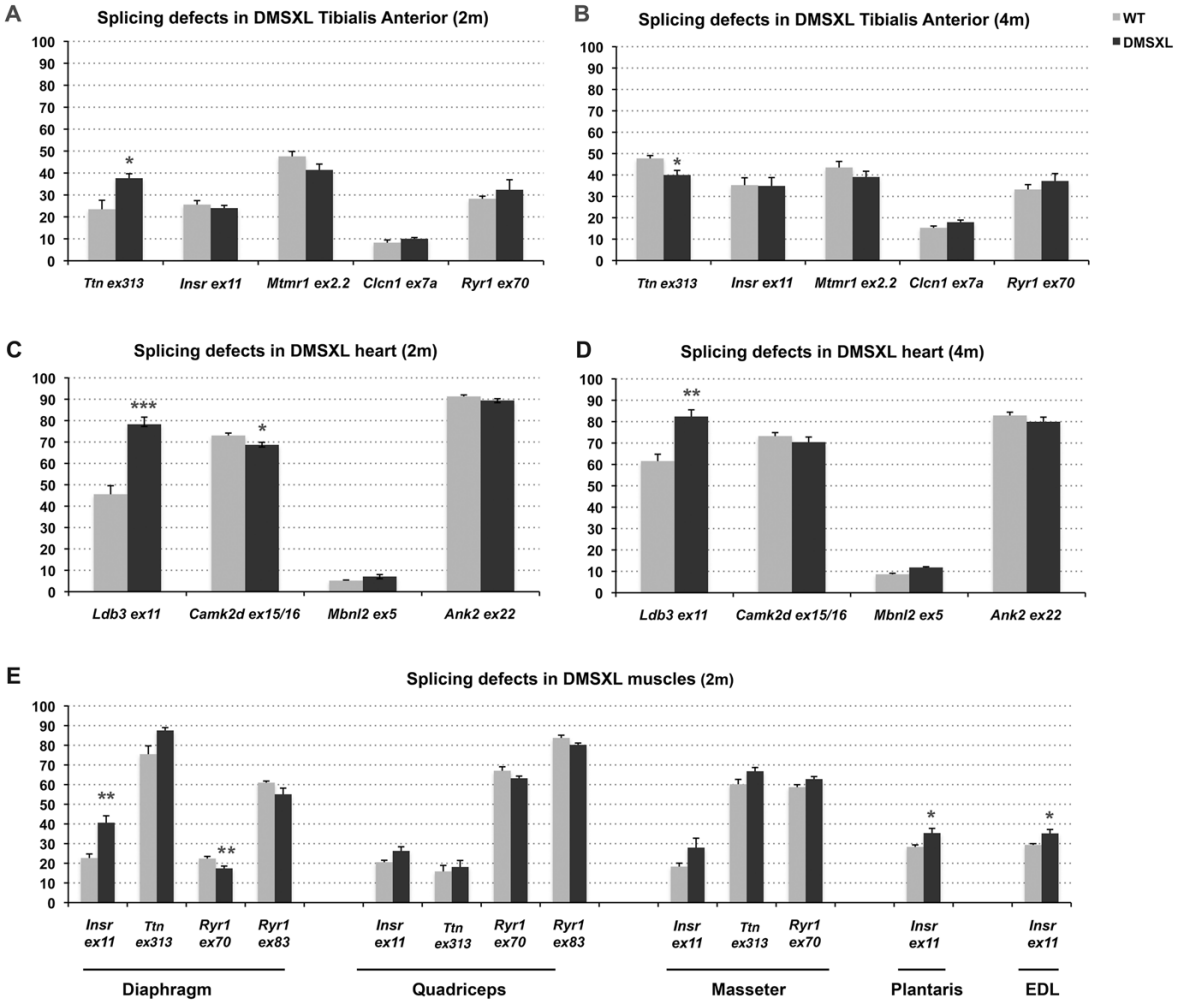
Splicing deregulation in DMSXL mice. Percentages of alternative exon inclusion in mRNA transcripts were studied by RT-PCR in 2- and 4-month-old mice in tibialis anterior (A–B) and heart (C–D), and in various muscles from 2-month-old mice (E). Results were compared between DMSXL (n = 6) and WT (n = 6). Each bar represents the mean of 6 biological replicates ± SEM. Differences in percentage of alternative exon inclusion between DMSXL and WT mice were determined to be statistically significant by Student's t test, * p<0.05, ** p<0.01, *** p<0.001. 2 m, 2- month-old; 4 m, 4-month-old.

A growing number of splicing defects were described in human DM1 tissues as well as in other DM1 mouse models [6]. We investigated some of the previously described splicing alterations in homozygous DMSXL tibialis anterior and heart at 2 and 4 months of age (Figure 6A–6D, Figure S5, Table 1). Splicing defects in DMSXL brain are described elsewhere (O. Hernandez-Hernandez et al., manuscript in preparation). Our study revealed some significant splicing abnormalities in DMSXL mice, while DM300 mice of the same age did not show obvious splicing abnormalities at these ages [24], [25]. However, missplicing in DMSXL remained mild and variable from one mouse to another. Some missplicing events were observed in most of the mice (such as *Ttn* exon 313 and *Ldb3* exon 11 missplicing in tibialis anterior and heart respectively) and were statistically significant (*Ttn* exon 313, p = 0.0113 and p = 0.0134 respectively in 2-month-old and 4-month-old tibialis anterior; *Ldb3* exon 11 p<0.0001 and p = 0.0017 in 2-month-old and 4-month-old hearts respectively, two-tailed Student's t test), while others were detected only in 30% of transgenic animals (such as *Insr* exon 11 in tibialis anterior). This inter-individual variability between the 5–6 mice studied per group resulted in a non-statistically significant tendency for some of the alternative exons presented in Figure 6. We then investigated various muscles at 2 months of age and observed significant splicing defects for *Insr* exon 11 in the diaphragm (p = 0.005, two-tailed Student's t test), plantaris (p = 0.0334, two-tailed Student's t test) and EDL (p = 0.0191, two-tailed Student's t test) as well as for *Ryr1* exon 70 in the diaphragm (p = 0.0071, two-tailed Student's t test) (Figure 6E, Figure S5). *Insr* exon 11 splicing defects were more variable in quadriceps and masseter.

**Table 1:** List of primers used for RT–PCR splicing analysis.

***Ank2 ex 22*** [52]	Forward	GAACGTGGTTCTCCGATTGT	***Mbnl2 ex 5*** [53]	Forward	ACCGTAACCGTTTGTATGGATTAC
(M/H)	Reverse	CGTCTCTGGGGGTATGTCAG	(M/H)	Reverse	CTTTGGTAAGGGATGAAGAGCAC
***Camk2d ex15/16*** [53]	Forward	TTGACAACTATGCTGGCTACGAG	***cTnT ex 4/5*** [52]	Forward	GTACGAGGAGGAACAGGAAG
(M/H)	Reverse	TTCACGTCTTCATCCTCAATGG	(H)	Reverse	CCAGCCTCCTCCTCCTCC
***Clcn1 ex7a*** [54]	Forward	CTTTGTAGCCAAGGTG	***Mtmr1 ex2.2*** [55]	Forward	CATGTTGAATGGTGTAAACAG
(M)	Reverse	ACGGAACACAAAGGCACTGA	(M)	Reverse	AATTATCCCCATGGCTCTGT
***Fxr1h ex15*** [52]	Forward	GATAATACAGAATCCGATCAG	***RyR1 ex70*** [55]	Forward	GACAATAAGAGCAAAATGGC
(M/H)	Reverse	CTGAAGGACCATGCTCTTCAATCAC	(M)	Reverse	CTTGGTGCGTTCCTGATCTG
***IR ex 11*** [25]	Forward	GAGGATTACCTGCACAACG	***RyR1 ex83*** [55]	Forward	CGAGAGGCAGAACAAGGCAG
(H)	Reverse	CACAATGGTAGAGGAGACG	(M)	Reverse	GGTCCTGTGTGAACTCGTCA
***Ldb3 ex11*** [53]	Forward	GGAAGATGAGGCTGATGAGTGG	***Serca1*** [53]	Forward	GCTCATGGTCCTCAAGATCTCAC
(M/H)	Reverse	TGCTGACAGTGGTAGTGCTCTTTC	(M)	Reverse	GGGTCAGTGCCTCAGCTTTG
***Mbnl1 ex 5*** [53]	Forward	GCTGCCCAATACCAGGTCAAC	***m-Ttn ex5*** [53]	Forward	GTGTGAGTCGCTCCAGAA
(M/H)	Reverse	TGGTGGGAGAAATGCTGTATGC	(M)	Reverse	CCACCACAGGACCATGTTATTTC

Name of genes, exons analyzed and primer sequences are indicated. RT-PCR analysis were performed in Muscle (M), Heart (H) or both (M/H).

However, one-way analysis of variance with Bonferroni post-test performed in each tissue and at each age revealed that only *Ttn* exon 313 at 2 months of age in tibialis anterior and *Ldb3* exon 11 at 2 and 4-months of age in heart remained significant. These data showed that the overall splicing defects detected in the DMSXL mice were mild, except for *Ldb3* exon 11 at 2 and 4 months of age and *m-Ttn* exon 313 at 2 months of age.

We looked if we could detect changes in the levels of CELF1 (or CUGBP1) in the heart of 2-month-old DMSXL mice by western blot analysis (Figure S6). A mild increase of CELF1 was observed only in one over 4 DMSXL mice tested, when compared with littermate wild-type controls (WT).

### DMSXL display high mortality, growth retardation, and lower fasting levels of IGFBP-3 and Insulin

**Figure 7:**
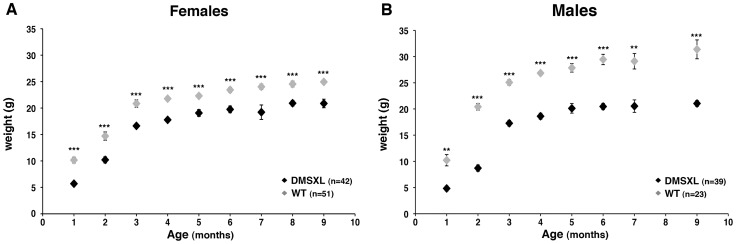
Weight monitoring and follow-up with age. Weight (in grams, g) was recorded for female (A) and male (B) DMSXL homozygotes (+/+) and WT littermate controls (WT). Differences in body weight between DMSXL and WT mice were determined to be statistically significant by Student's t test, ** p<0.01, *** p<0.001.

Some hemizygous DMSXL mice carrying very large expansions of >1000 CTG displayed growth retardation, however most of them did not show an obvious phenotype. This observation indicates that the dramatic increase in CTG repeat size in DMSXL mice is not sufficient to overcome the low expression of the mutant *DMPK* RNA in these animals. It is noteworthy that we observed a 31% decrease of *DMPK* RNA levels in DMSXL heart compared to DM300, carrying about 500 to 600 CTG repeats (Figure S7). Homozygous DMSXL exhibit severe growth retardation (more severe than DM300 mice). Mouse weight monitoring showed that during the first month of life, homozygous DMSXL mice were much smaller than their WT littermates (about 50%). After 2 months of age, DMSXL females and males caught up in weight (70–80% for females, and 60–70% for males; Figure 7A and 7B). Male mice reached a plateau at 3.5 months. We observed a high frequency of death before weaning in litters from breeding between hemizygous DMSXL. We estimated the mortality about 60% before 1 month of age for the homozygous DMSXL (counting the progeny at one month of age revealed 278 hemizygous, 68 homozygous and 166 WT mice; this showed a clear deficit in homozygous of about 60%, with regards to the WT frequency, p<0.0001, Chi-square test). After weaning the mortality was about 5% between 1 and 5 months.

**Figure 8:**
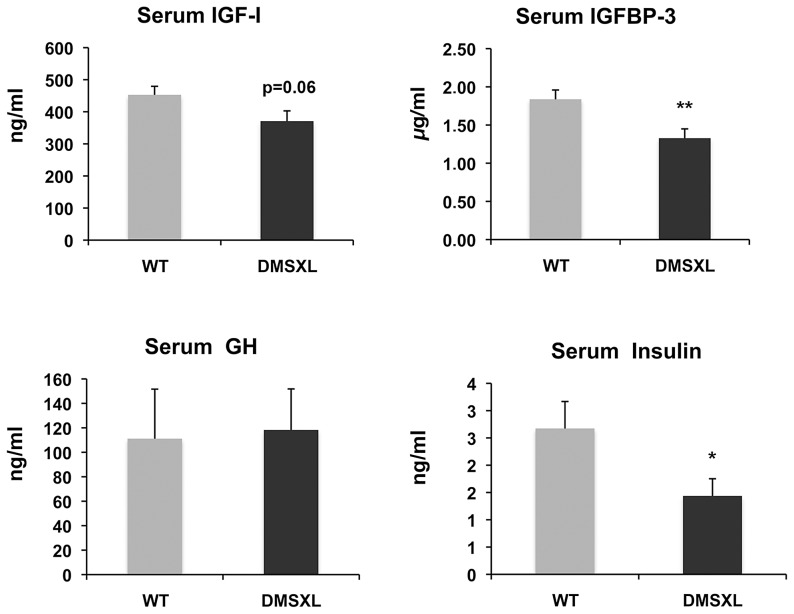
Circulating levels of growth-related signaling proteins after fasting. Significant decreases of IGFBP-3 and insulin levels were observed in 4-month-old homozygous DMSXL. A decrease of IGF-I was also observed but did not reach statistical significance. GH levels were variable but similar between DMSXL and WT. Data are presented as means ± SEM (* p<0.05, ** p<0.01, Student's t test or Mann-Whitney test), n = 12 biological replicates per group.

To gain insight into the mechanisms behind the growth phenotype of DMSXL mice, ELISA and Western Ligand Blot analyses were performed to quantify Insulin-like growth factor I (IGF-I), IGF binding protein-3 (IGFBP-3, the predominant IGF carrier protein in circulation), Growth Hormone (GH) and Insulin in 4-month-old WT and DMSXL females (n = 12 of each genotype). Serum levels were measured after overnight fasting (Figure 8). Serum IGFBP-3 level was significantly reduced by 24% in DMSXL mice compared to age and sex-matched WT controls (p = 0.0083, two-tailed Student's t test). Serum IGF-I levels showed a marked tendency towards reduction in DMSXL mice (p = 0.0632, two-tailed Student's t test), while GH levels, although very variable, were similar between DMSXL and WT mice. Fasting serum Insulin level was also dramatically reduced by about 46% (p = 0.0304, Mann-Whitney test).

### DMSXL exhibit abnormal skeletal muscle histological features and increased proteasome activity

**Figure 9:**
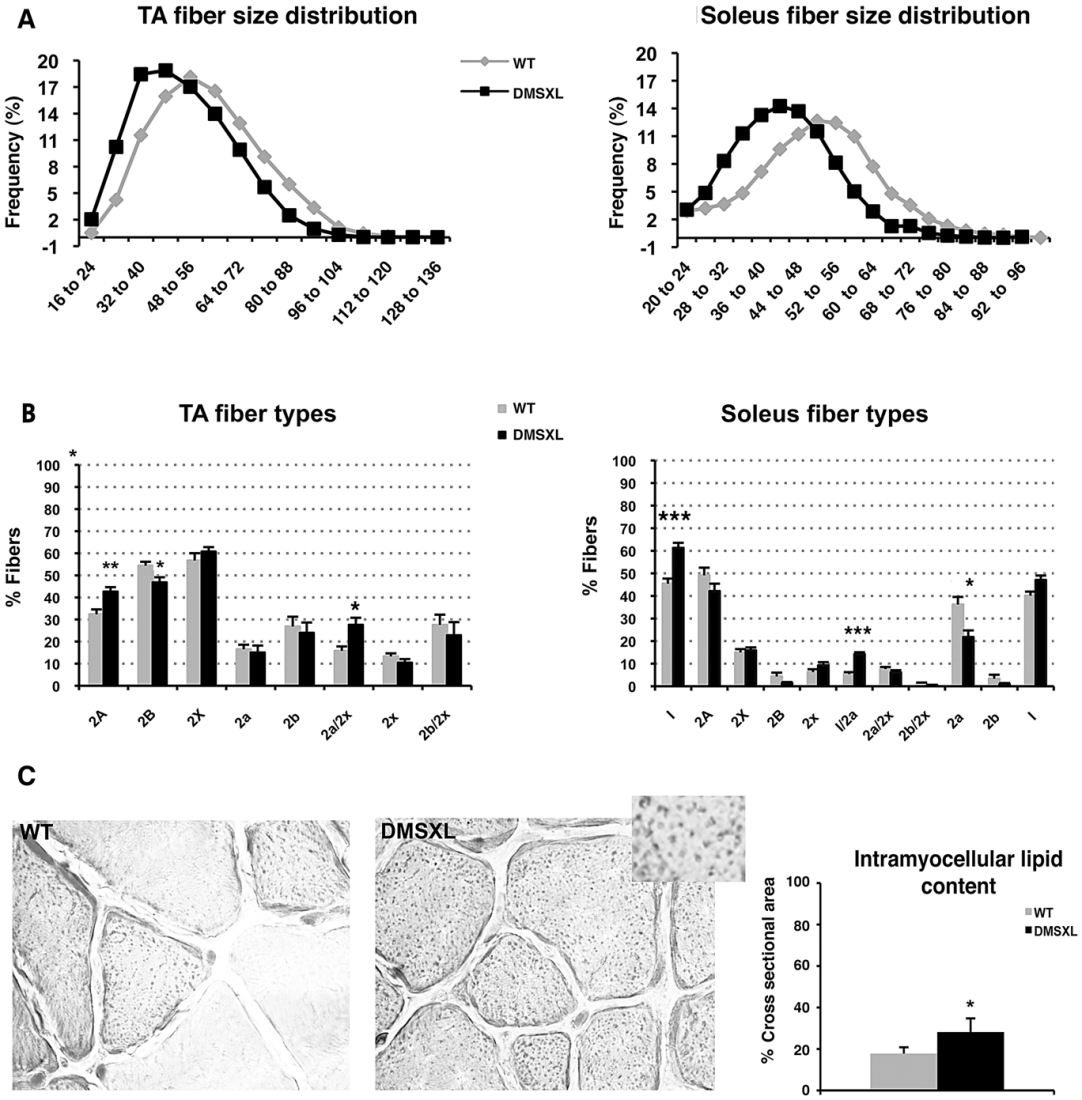
Histological abnormalities in DMSXL muscles. (A) Fiber size (cross-sectional area, pixels) was measured in tibialis anterior (TA) and soleus muscles from 4-month-old female WT (grey line) and DMSXL (black line) mice, n = 3 biological replicates in both groups. (B) Fiber types were determined in TA and soleus muscles from WT (grey histograms) and DMSXL (black histograms) mice, n = 3 biological replicates in both groups. (C) Oil Red O staining of muscles section (TA) showed intramyocellular lipid accumulation in DMSXL mice. Data are presented as means ± SEM (* p<0.05, ** p<0.01, *** p<0.001, Chi-square or Student's t tests), n = 4–5 in both groups.

To characterize the muscle phenotype of DMSXL mice, the number and size of muscle fibers were analyzed in both tibialis anterior and soleus muscles from 4-month-old mice. Fiber size was measured and classified according to the cross-sectional area (CSA, Figure 9A). In tibialis anterior, the number of small size fibers was significantly increased in DMSXL mice when compared to age-matched WT mice (p = 0.03, Chi-squared analysis). A 31% reduction in the mean muscle fiber CSA was found in the DMSXL mice when compared to the WT mice, but there was no change in the total number of fibers (2869±200 vs 2794±150). A significant (p = 0.005, Chi-squared analysis) decrease in the number of large fibers was also observed in DMSXL soleus muscles (with no change in the total number of fibers (745±30 vs 795±80). Myosin heavy chain (MHC) content analysis showed that the fibers with reduced size were not restricted to any specific type of fiber (data not shown). However a significant increase in the percentage of type 2A and 2a/x fibers (10%, p = 0.0063 and 12%, p = 0.0133, respectively, two-tailed Student's t test) was measured in the tibialis anterior of DMSXL mice when compared to WT mice (Figure 9B). In contrast, the percentage of type 2B was significantly reduced by 8% (p = 0.0291, two-tailed Student's t test). In soleus, the percentage of type 1 and 1/2a fibers was significantly increased by 12% (p = 0.0008, two-tailed Student's t test) and 7% (p<0.0001, two-tailed Student's t test) respectively in DMSXL mice (Figure 9B).

Besides a decrease in the mean muscle fiber CSA and an increase in the number of oxidative fibers, the lipid content as well as the fibrosis were examined in 4-month-old DMSXL muscles. Red Sirius staining showed that the level of fibrosis was not increased in DMSXL muscles when compared to WT (data not shown). In contrast, fatty content assessed by Oil Red O staining showed that intramyocellular lipid accumulation is increased by 59% in the tibialis anterior of DMSXL mice (p = 0.0220, two-tailed Student's t test) (Figure 9C). Finally, some centronucleated fibers were observed in DMSXL muscles, however at very low frequency (data not shown).

**Figure 10:**
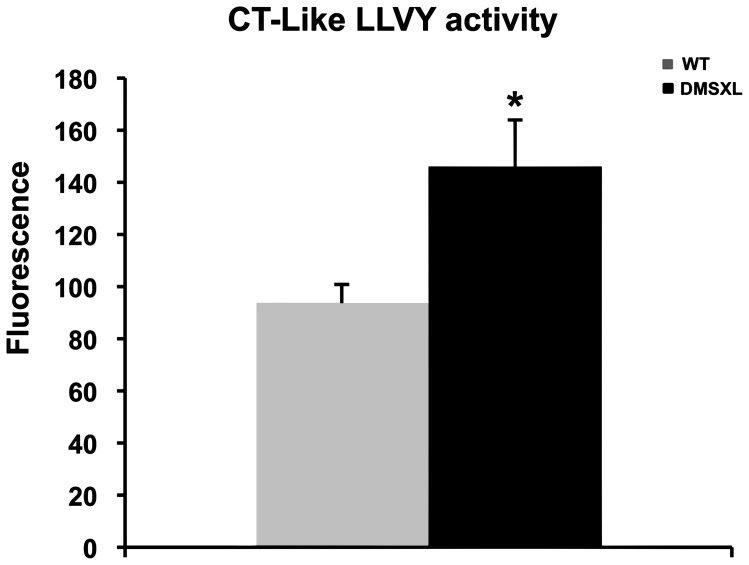
Proteasome activity in DMSXL mice. Chymotrypsin-like activities of the proteasome were assayed using the fluorogenic peptides LLVY-AMC in 4-month-old female WT and DMSXL mice. Each group contained ten animals and each animal was assayed in triplicate. Histogram bars represent the average activity ± SD for each group (* p<0.05, Student's t test).

Proteasome peptidase activities were measured in the tibialis anterior using fluorogenic peptide substrates. All assays were performed in the absence or presence of the proteasome inhibitor MG132, to distinguish proteolysis by the proteasome from other co-purifying proteases. As shown in Figure 10, the chymotrypsin-like proteasome peptidase activity was increased by 38% in the DMSXL mice, when compared to WT (p = 0.0138, two-tailed Student's t test). No significant differences were observed for the trypsin-like and caspase-like activities (data not shown) between the two groups. The activities were completely inhibited when MG132 was added to the reaction mixture in both DMSXL and WT mice, indicating that the activities measured were specific for the proteasome.

### DMSXL show decreased muscle strength *in situ* and *in vivo*

**Figure 11:**
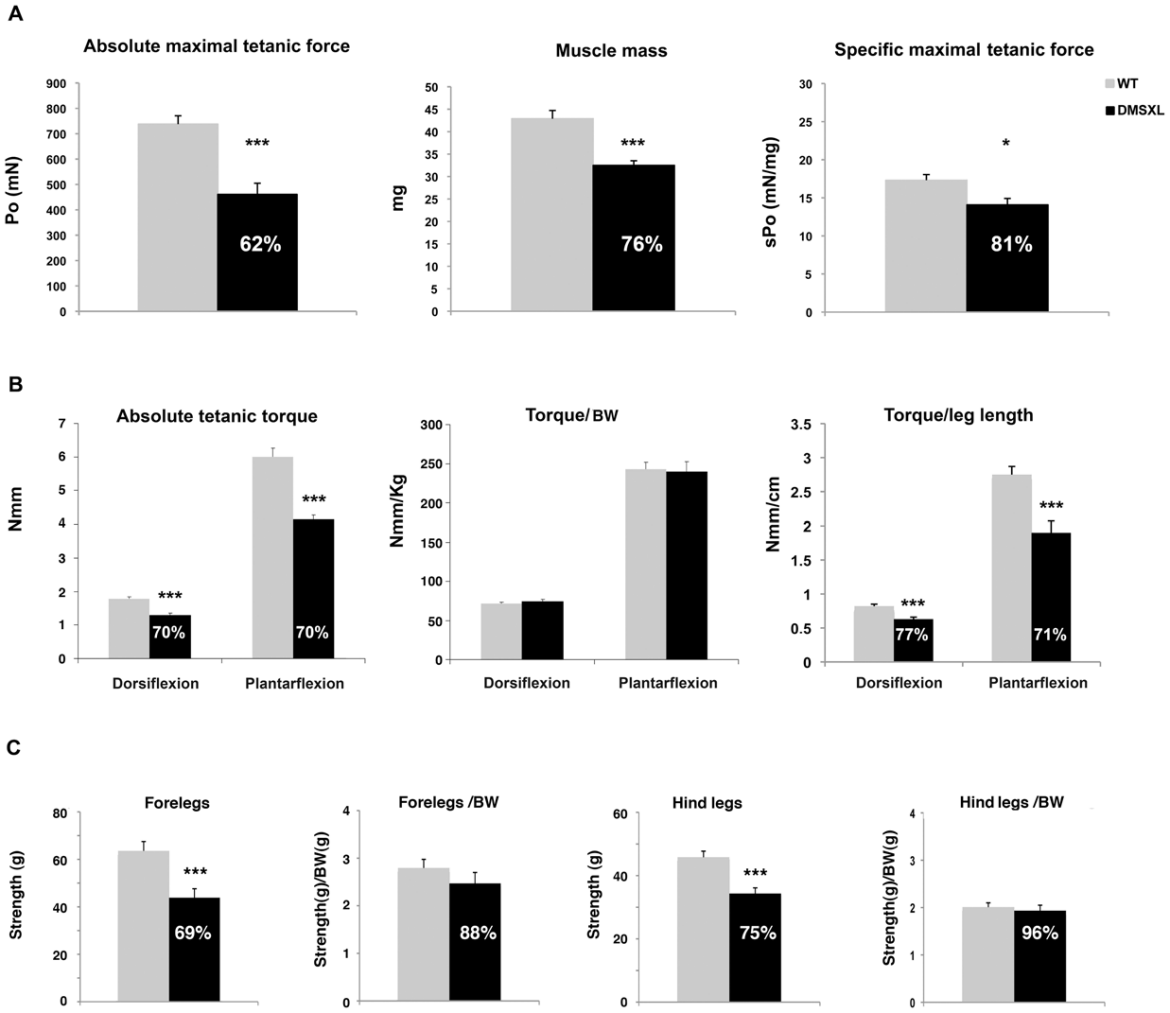
Muscle force and strength measurement in DMSXL mice. (A) Absolute maximal tetanic force was measured *in situ* for WT and DMSXL female mice between 4 and 6 months of age, as well as muscle mass. The specific maximal tetanic isometric force represented the absolute maximal tetanic isometric force normalized to the muscle mass, n = 8 biological replicates in both groups. (B) Absolute tetanic torque dorsiflexion and plantarflexion in WT and DMSXL mice were determined using a home built dynamometer. Absolute tetanic torque normalized to mouse weight or leg length are also shown, n = 8 for both groups. (C) Grip strength was measured with a grip dynamometer in WT and DMSXL forelegs and hind legs and normalized to body weight. n = 22 biological replicates in both groups. Data are presented as means ± SEM (* p<0.05, ** p<0.01, ANOVA, Student's t test). The mice were analyzed between 4 and 5 months of age. BW: Body weight.

The contractile properties of isolated tibialis anterior and soleus muscles of 4-month-old animals were measured by *in situ* and *in vitro* analysis, respectively. A 38% reduction of the maximal tetanic isometric force (Po) was measured in the tibialis anterior of female DMSXL mice (n = 9, p = 0.0002, two-tailed Student's t test), whereas the tibialis anterior mass is only decreased by 24% (Figure 11A). The significant 19% decrease of the specific Po (sPo, p = 0.0296, two-tailed Student's t test), after muscle mass normalization, indicates that the reduced force observed in the tibialis anterior of the DMSXL mice is not solely explained by the decrease in muscle mass. Additional mechanisms related to the large CTG expansion may alter the contractile properties of the tibialis anterior. In contrast, no decrease in the specific Po was observed in the soleus of female DMSXL mice (n = 9), despite a significant 23% decrease in the maximal tetanic isometric force (p = 0.0005, two-tailed Student's t test), most likely as the consequence of muscle atrophy (29% decrease of the soleus mass, data not shown).

The maximal tetanic torque of both ankle dorsi- and plantarflexion was determined *in vivo* in 8 WT and 8 DMSXL anesthetized females at 4 months of age. Ankle dorsiflexion and plantarflexion torques developed by the DMSXL mice were about 30% lower than those of WT mice (Figure 11B). When normalized to body weight, the differences in ankle torque between DMSXL and WT mice vanished. Since the lower body weight in DMSXL mice may not only be related to reduced muscle mass, we also normalized the ankle torques to the leg length of the animals. The leg length was slightly but significantly diminished in DMSXL compared to WT mice (2.06±0.1 cm versus 2.19±0.1 cm, respectively, p<0.0001, ANOVA. Not shown). The dorsi- and plantarflexion torques normalized to leg length were respectively reduced by 23% and 29% in DMSXL compared to WT mice (Figure 11B). We measured *in vivo* the half-relaxation time, the late relaxation time and the myotonic index of the maximal tetanus of ankle dorsi- and plantarflexors. Only a non-significant trend for a slightly longer late relaxation time appeared for DMSXL mice using this technique. Using EMG, we could detect mild myotonia in leg muscles after needle insertion (data not shown). However, the myotonic discharges were small and the mice tested showed, in our hands, myotonia of grade 1 according to the scale reported by Wheeler *et al* [28].

Global limbs muscle strength was measured using a Grip dynamometer for both forelegs and hind legs from 4-month-old DMSXL and WT littermate females (Figure 11C). 4-month-old DMSXL mice showed significant decreases in limb strength in forelegs (31%, p = 0.0008, two-tailed Student's t test) and hind legs (25%, p<0.0001 two-tailed Student's t test), whereas 5-month-old DM300 female mice did not show significant limb strength difference relative to WT controls [22]. However, these differences were no longer significant after body mass normalization.

### DMSXL mice display reduced motor performances

**Figure 12:**
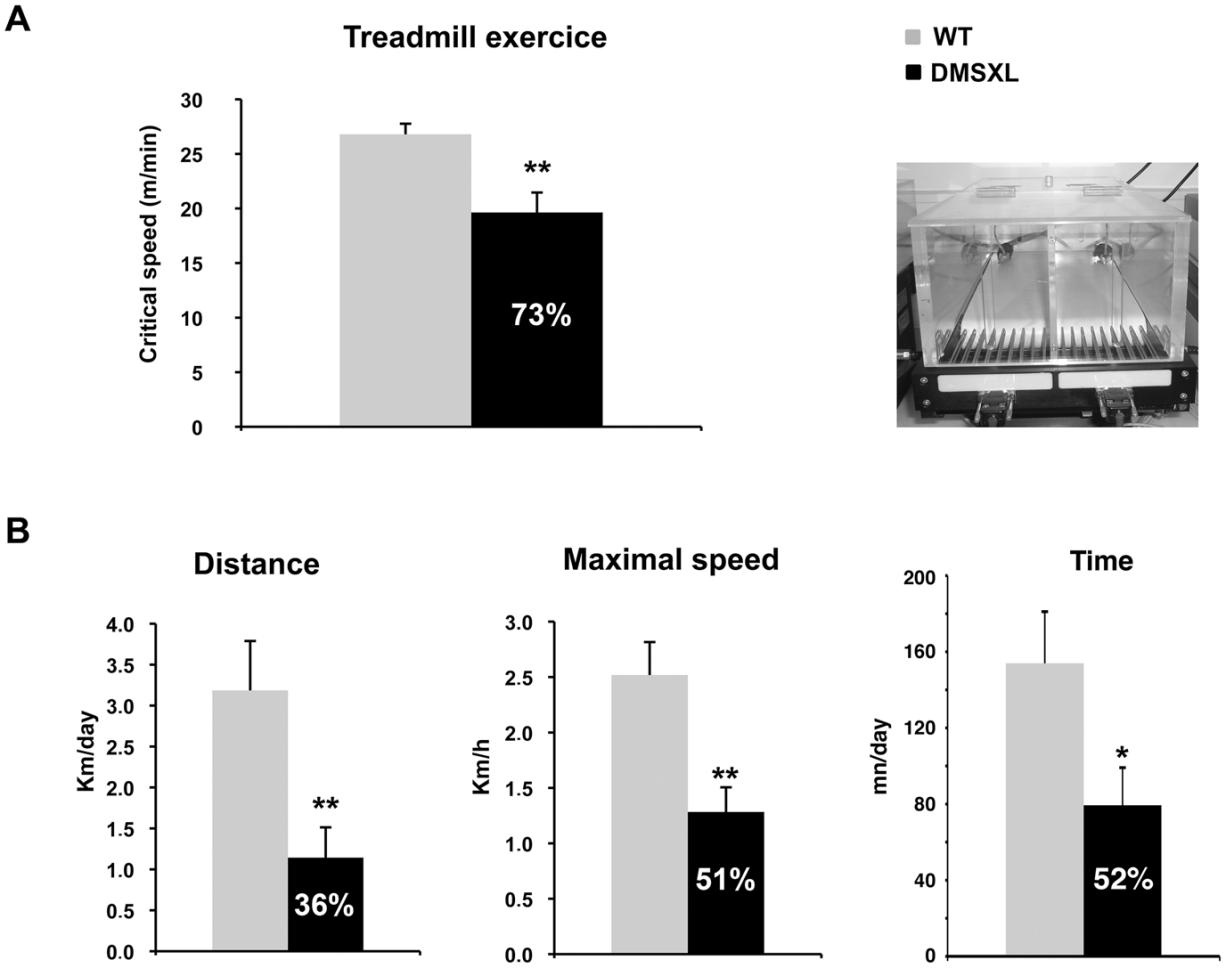
Motor performance of DMSXL mice. (A) Treadmill test was performed on WT and DMSXL female mice between 4 and 6 months of age. The Critical Speed was calculated from the slope of the regression line, plotting the distance versus the time to exhaustion, n = 9 for both groups. (B) The wheel running test was performed on WT and DMSXL mice between 4 and 6 months of age. The mice were followed over a period of 10 days. The distance, the maximal speed and the time spent running are shown, n = 14 biological replicates for both groups. Data are presented as means ± SEM (* p<0.05, ** p<0.01, Student's t test or Mann-Whitney test).

Global and non-invasive approaches were used to evaluate the disease-related impairment of the muscular function in 4–5-month-old DMSXL mice. Physiologic performances were assessed in WT and DMSXL females by treadmill exercise and by the wheel test (Figure 12).

In the treadmill test, the mice were subjected to a running paradigm to determine the time to exhaustion and to define a parameter of motor performance, the critical speed (Csp). After an acclimatizing period, the mice were evaluated on the treadmill. A significant 27% decrease of the Csp was measured in the DMSXL mice when compared to WT controls, indicating that the motor performances of the DMSXL mice are impaired (p = 0.0031, two-tailed Student's t test, Figure 12A).

The wheel test reveals animal's voluntary capacity for running (*i.e.* the mice run voluntarily during the night on a wheel in their home cage, Figure 12B). The evaluation was done over a period of 10 days. We observed a significant 64% decrease in the distance that DMSXL mice ran per night, when compared to WT mice (p = 0.0026, Mann-Whitney test). The maximal running speed of DMSXL mice was significantly decreased by 49% (p = 0.0028, Mann-Whitney test, Figure 12B). The time to accomplish this distance and the average speed were also significantly reduced by 48% (p = 0.0389, two-tailed Student's t test, Figure 12B) and 40% (p = 0.0057, Mann-Whitney test, data not shown), respectively, for the DMSXL mice when compared to WT controls.

Similar results were obtained on males, although the results were more variable in these tests due to a lower cooperation of both WT and DMSXL males.

## Discussion

DMSXL mice carrying more than 1000 CTG repeats embedded in their human genomic context have the advantage to express the *DMPK* gene under the regulation of its own promoter and of at least some of its regulatory elements, probably carried by the large transgene. This allowed expression of the human *DMPK* with a pattern similar to the patterns of the murine *Dmpk* gene in mice and the *DMPK* gene in human tissues (Figure 2). Interestingly, we observed different levels of expression between different muscles for both the *DMPK* transgene and the murine *Dmpk* gene (Figure 2). Differences in the production of toxic RNA could explain, at least in part, why some skeletal muscles are more affected than others in DM1 patients. The levels of human *DMPK* transcripts were generally lower than the levels of murine *Dmpk* transcripts in DMSXL mouse tissues. We previously observed that independent transgenic lines carrying about 300 CTG repeat expansions expressed less *DMPK* transcript than transgenic mice carrying short 20 CTG repeats. This indicated that increasing CTG repeat length alters the production of transcripts in transgenic mice [24]. This was confirmed by the 31% decrease of *DMPK* expression in mice with >1000 CTG, in comparison with mice carrying 500–600 CTG repeats (Figure S7). In addition, we cannot exclude different efficacies of the mouse regulatory factors (transcriptional or post-transcriptional regulatory factors) towards human DNA or RNA sequences. This could explain in part why the human *DMPK* transgene is expressed at low levels in some mouse tissues. However, this is not the case in brain in which we observed higher levels of transgenic *DMPK* compared to the murine *Dmpk* transcripts. The higher *DMPK* expression in mouse brain could be mediated by the influence of the mouse genomic sequence located near the integration site. However, we do not favor this hypothesis because the relative high expression in brain is also observed in other independent transgenic lines carrying expanded or normal repeats (data not shown). Furthermore, all our different mouse lines express the transgene, suggesting that the large transgenic fragment used carries sequences that allow *DMPK* to be “isolated” from the murine genomic environment [24]. Indeed, an insulator element has been described downstream of the CTG repeat and it is included in our construct [29]. The promoter for the *DMPK* antisense has been located in this regulatory region [16] and we demonstrated in this study that antisense *DMPK* is expressed in various DMSXL tissues, as well as in various human control tissues. Although it has been proposed that antisense transcripts are converted in 21-nucleotide fragments [26], we could detect antisense RNA after the CAG repeats in 3′ and also antisense RNA ribonuclear foci, showing that antisense transcription extends across the CAG repeat. We observed similar levels of transcripts in 5′ and 3′ of the repeat in human tissues (except in brain), but the levels of antisense were lower after the repeat in tissues from mice carrying large expansion, suggesting that transcription through large repeats is affected or that RNA carrying CAG expansion are less stable.

As expected sense RNA transcripts carrying CUG repeats formed numerous foci in a large variety of tissues, which reflect the ubiquitous expression of *DMPK* and the potential toxic effect of the mutation in different tissues. More foci were detected in DMSXL tissues compared with DM300, which suggests that longer CTG repeats favor foci formation. Furthermore, when comparing the number of foci in heart tissues between homozygous and hemizygous mice from the same litter, we observed higher levels of nuclei containing foci in the homozygote. This suggests that higher levels of RNA favor foci formation. However, other parameters and factors govern foci accumulation, such as MBNL1 or hnRNP H proteins, whose levels can vary between different tissues. The complex relationship between CTG repeat lengths, levels of CUG/CAG-containing RNA and levels of proteins involved in foci formation in different tissues requires further investigation. In DMSXL heart and muscles, we observed that they co-localized with and titrated, at least in part, MBNL1 and MBNL2 proteins. Surprisingly, we also observed nuclear foci formed by antisense CAG-containing transcripts. CAG RNA foci have already been described in CAG repeat diseases but have never been reported in DM1 [26]. The role of the *DMPK* antisense in DM1 pathology is unclear but the formation of nuclear foci and the possibility of being translated into homopolymeric peptides by RAN translation increase the possible levels of toxicity. Very interestingly, RAN translation and polyglutamine aggregates have been observed in DMSXL cardiac tissues [17]. Although we observed co-localization of the antisense foci with MBNL1 in human DM1 heart, we did not see clear co-localization with MBNL1 or MBNL2 in DMSXL heart and skeletal muscles, probably due to the low levels of antisense transcripts in the transgenic mice.

Splicing regulatory defects has been well documented in DM1 patients and in various mouse models, although only a few of them have been shown to be causative of DM1 symptoms [7]. Alternative splicing dysregulation can also be a secondary event, reflecting the physiological state of a tissue, as recently demonstrated in mouse muscles [30]. Foci formation and abnormal splicing events recently emerged as molecular readouts in the preclinical assessment of therapeutic strategies for DM1 [6], [31]. We then looked for splicing defects in muscle and heart from DMSXL mice and tested previously described candidates. In contrast with the DM300 mice (carrying about 500 CTG repeats) that only showed *Insr* exon 11 splicing alteration at 10 months of age, we could detect several missplicing events in DMSXL muscles and heart at 2 and 4 months of age. These splicing defects were moderate and variable from one mouse to another (Figure 6), probably due to low expression of the transgene even in homozygous mice. However, our data showed that increasing the CTG repeat length in our mouse model induced more splicing defects (missplicing events in brain are reported elsewhere; O. Hernandez-Hernandez et al., manuscript in preparation) but only a few of them can be used as biomarkers in therapeutic strategy targeting expanded RNA. The result obtained with *mTtn* ex5 was puzzling. We observed an increase in exon 5 inclusion in 2-month-old DMSXL mice, as observed in other DM1 mouse models, and in agreement with a return to a fetal splicing profile [32]. At 4 months of age, WT mice showed an increase of exon 5 inclusion relative to 2 months. No such increase was observed in DMSXL, which showed a lower inclusion of exon 5 compared to WT mice. This could reflect either a direct lack in splicing regulation in DMSXL or a different muscle physiological state.

Homozygous DMSXL showed a stronger phenotype and were smaller than homozygous DM300 mice (at 3 month of age, weights were 63% and 79% respectively for DMSXL males and females and 84% and 91% for DM300 males and females). As previously reported, mice carrying a normal repeat of 20 CTG were normal [24]. Taken together, these results show that the decrease in weight is linked to the CTG repeat expansion, rather than to the over expression of the transgenes carried by the transgenic mice. Very interestingly, Charizanis *et al* recently reported that *Mbnl2* knockout mice are also smaller at weaning strengthening the link between toxic RNA, depletion of MBNL2 and growth [33]. Growth hormone and IGF-1 signaling pathways are known to play key roles in regulating body growth, tissue remodeling, skeletal muscle growth and differentiation and homeostasis of the adult muscle tissues [34]. Frequent endocrine alterations have been reported in myotonic dystrophy (DM) [35], as well as glucose intolerance and hyperinsulinemia [36]. IGF-1 is reduced in DM1 patients. Although the cause of reduced levels of IGF-1 is not clear, it was proposed to be related in part to disturbances in the DM1 hypothalamo-pituitary-adrenal axis [37]. Therefore we measured circulating fasting levels of IGF-1, IGFBP-3, GH and insulin. Interestingly, we found significant decreases in the basal levels of Insulin and IGFBP-3 and a tendency for a decrease of IGF-1. The level of GH was similar between DMSXL and WT controls. However, it is very difficult to assess GH levels due to their pulsatile secretion patterns. Since IGF-I and IGFBP-3 are regulated by GH, they are frequently used as indirect measures of GH action. The results obtained in DMSXL mice suggest deficits of GH signaling and could explain, at least in part, the growth defect observed in these mice. DM300 mice showed abnormal insulin response to glucose overload but no fasting decrease of basal insulin, in contrast to DMSXL [25]. These observations confirm a link between abnormal insulin level and the CTG repeat size. However, the endocrine abnormalities observed in our transgenic mouse models do not fully mirror those observed in DM1 patients and demonstrate the limitation of mouse models to recreate some disease symptoms.

DMSXL mice showed a decrease in the mean muscle fiber CSA, an increase in the number of oxidative fibers, and higher intramyocellular lipid accumulation. Interestingly, fatty infiltration and greater intramyocellular lipid contents in DM1 muscles were reported in several studies [1], [38], [39]. These abnormal muscle histological features were associated with a significant 19% decrease of the specific Po (sPo) in the tibialis anterior, suggesting that the large CTG expansion may alter the contractile properties of this muscle. DMSXL mice showed also decreased maximal tetanic torque of the ankle dorsi- and plantarflexion, decreased muscle strength in grip test, and reduced performance in the treadmill and wheel tests. For these physiological tests, it is difficult to know if the reduced performances are only due to the overall reduction in size of the DMSXL mice. However, the significant decrease of the specific force in DMSXL tibialis anterior (normalized to muscle mass), not observed in DM300 of the same age [22], suggests that additional mechanism related to the large CTG expansion may alter the properties of this muscle. Myotonia could be detected using EMG but remained very mild. Splicing defects for *Clcn1* were difficult to observe and again were very variable between mice (Figure S5). We estimate that myotonia is too mild in these mice to be used as an outcome measure for preclinical tests. A peripheral neuropathy has been also observed in DMSXL mice [40]. Previous analysis of DM300 mice carrying about 550 CTG repeats, showed that progressive muscle weakness occurred between 3 and 10 months of age and was associated with the activation of the ubiquitin–proteasome pathway [22]. DMSXL also show a significant increased proteasome activity already detected at 4 months of age suggesting that this proteolytic pathway could also participate in the physiopathological remodeling of the muscle.

In conclusion, DMSXL mice carrying >1000 CTG repeats express *DMPK* transcripts in a variety of tissues due to the ubiquitous activity of the *DMPK* gene own promoter. Foci accumulation has been observed in numerous tissues suggesting a multisystemic toxic effect of these mutant transcripts. We also demonstrated that antisense RNA carrying CAG repeats are transcribed in various tissues and can form nuclear foci. They can also be translated in polypeptides by RAN translation as recently demonstrated [17]. Therefore by using large genomic sequences with the *DMPK* gene carrying a very large repeat expansion, we recreate, at least in part, some of the complex molecular features of DM1. Although the mild splicing defects observed in DMSXL constitute a limitation of this mouse model, this mouse line can be used to investigate various aspects of the multisystemic consequences of the production of toxic expanded RNA in various organs. It can also be used for preclinical testing of multisystemic therapeutic strategies in order to evaluate their efficacy in various tissues. The *DMPK* transgene is expressed very early during development and in DMSXL neonates (data not shown). The growth retardation observed after birth and the high perinatal mortality suggest that DMSXL mice may also reproduce neonatal features of CDM, which remain to be explored.

## Materials and Methods

### Animals

The DMSXL mice (>90% C57BL/6 background) carried 45 kb of human genomic DNA cloned from a DM1 patient as previously described [18]. Transgenic status was assayed by PCR [23]. Housing and handling of mice were performed in accordance with the guidelines established by the *French Council on animal care “Guide for the Care and Use of Laboratory Animals”: EEC86/609* Council Directive - Decree 2001-131. DM300 mice originally obtained in our laboratory, carried between 400 and 600 CTG repeats in this study. The DMSXL mice derived from the DM300 after large expansion events carried between 1000 and 1600 CTG repeats in this study, with a mean of 1250+/−110 CTG for the splicing studies and 1300+/−130 CTG for the physiological tests in the homozygous mice.

### RNA isolation

Tissues were homogenized in Trizol (Life Technologies) using a tissue lyser (Retsch MM400): twice for 2 min 30 sec at 23 pulsations/sec with 2 stainless 5 mm steel beads (Qiagen). After chloroform extraction, the aqueous phase was mixed with 70% ethanol and transferred to a Spin Cartridge of the PureLink RNA Mini Kit (Ambion by Life Technologies). Total RNA was extracted according to the manufacturer's protocol. A PureLink DNase step was inserted in the protocol, after binding of the RNA to the column as recommended. The quantity of RNA was determined by absorbance at 260 nm with a Nanodrop and the quality was verified on an agarose gel.

### RT–PCR

Before RT-PCR, RNA was treated with DNase (Qiagen) at room temperature for 15 min. The enzyme was inactivated at 70°C for 10 min. From 1 µg of RNA, cDNA was synthesized using Superscript II Reverse Transcriptase (Life Technologies) when using random hexamer primers, or using Superscript III Reverse Transcriptase (Life Technologies) when using strand-specific primers, according to manufacturer's protocols. The cDNA was digested with RNase A for 20 min at 37°C. Transcript levels were determined in human tissues using FirstChoice Human Total RNA Survey Panel (Ambion by Life Technologies). Total RNA from diaphragm was from BioChain. For RT-PCR analysis of alternative splicing, all samples were normalized to *18S* ribosomal transcripts. Following electrophoresis through agarose gels stained with ethidium bromide, the density of each PCR band was quantified using Quantity One 1D Analysis Software (Bio-Rad). The inclusion ratio of alternative exons in each animal was determined using 2 RT experiments and two replicate PCR amplifications to minimize experimental variation. The percentage of exon inclusion was calculated as [exon inclusion band/(exon inclusion band+exon exclusion band)]×100.

See Table 1 and Table 2 for primer sequences.

**Table 2:** List of primers used for expression analysis.

	Oligo for RT	Random hexamers
***DMPK*** ** sense (random priming)**	Forward	GGAGAGGGACGTGTTG
	Reverse	CTTGCTCAGCAGTGTCA
	Oligo for RT	Random hexamers
***Dmpk*** ** sense (random priming)**	Forward	GGAAGAAAGGGATGTATTA
	Reverse	CTCAGCAGCGTTAGCA
	Oligo for RT	CGACTGGAGCACGAGGACACTGACTTGCTCAGCAGTGTCA
***DMPK*** ** sense (specific RT)**	Forward	CGACTGGAGCACGAGGACACTGA
	Reverse	GGAGAGGGACGTGTTG
	Oligo for RT	CGACTGGAGCACGAGGACACTGAGACCATTTCTTTCTTTCGGCCAGGCTGAGGC
***DMPK*** ** antisense (specific RT in 5′)**	Forward	GGAGCACGAGGACACTGA
	Reverse	TGCGAACCAACGATAG
	Oligo for RT	CGACTGGAGCACGAGGACACTGACGCCTGCCAGTTCACAACCGCTCCGAGCGT
***DMPK*** ** antisense (specific RT in 3′)**	Forward	GGAGCACGAGGACACTGA
	Reverse	CCTTCGAGCCCCGTTCGC
	Oligo for RT	Random hexamers
**18S (random priming)**	Forward	CAGTGAAACTGCGAATGG
	Reverse	CGGGTTGGTTTTGATCTG

Name of RNA, type of reverse transcription and primer sequences are indicated. In 5′ and in 3′ refer to the position of the amplicon with regards to the CAG repeat in the antisense transcript.

### Quantitative RT–PCR

The transcripts were amplified in a 7300 Real Time PCR System (Applied Biosystems) using Power SybrGreen detection (Life Technologies). Annealing temperatures and sample dilutions were optimized for each amplicon. *DMPK* mRNA levels were calculated relative to *18S* transcripts. Oligonucleotide primer sequences are described in Table 2. For qRT-PCR, we used standard curves established with mouse tissues expressing the tested genes or a plasmid carrying the amplicon. In comparative studies, the number of molecules of each plasmid was carefully determined by O.D. and qRT-PCR in order to use the same number of molecules in all the experiments. The reverse transcription efficiency for each gene and each primer set was verified using increasing amounts of RNA input. Samples were performed in triplicate and experiments were repeated twice. *18S* was generally used as internal control as well as mouse *Dmpk* gene, when comparing expression levels between genotypes in the same tissue. RNAs from 3 sex- and aged- matched mice were pooled for qRT-PCR experiments.

### FISH

Sense ribonuclear inclusions were detected with a 5′-Cy3-labeled (CAG)_5_ PNA probe as described [24]. For antisense ribonuclear inclusions detection we used a 5′-Cy3-labeled (CTG)_5_ PNA probe using the same protocol. For the simultaneous FISH detection of both sense and antisense foci, we first performed hybridization using the 5′-Cy3-labeled (CAG)_5_ probe. After 3 washes in 1XPBS for 2 min at room temperature, the slides were subsequently hybridized with a green 5′-Alexa 488- (CTG)_5_ probe. Co-localization experiments with MBNL1 and MBNL2 were performed as previously described [24]. We used MBNL1 MB1a and MBNL2 MB2a antibodies that were kindly provided by Ian Holt [41]. Human DM1 heart samples were obtained from MYOBANK-AFM (Paris, France). The DM1 patient was diagnosed with ∼170 CTG. Images were acquired using the Zeiss Apotome, then treated using the ImageJ software (Rasband, W.S., ImageJ, U. S. National Institutes of Health, Bethesda, Maryland, USA). The number of foci, their nuclear localization and the co-localization with MBNL proteins were checked in 3D using the Imaris software (BITPLANE).

### Western blotting

Proteins were extracted by mechanical homogenization in lysis buffer (0.125 M Tris-HCl pH 6.8, 4% SDS, 10% glycerol) containing a complete protease inhibitor cocktail (Roche) and 1 mM PMSF (Sigma). Proteins were denatured 5 min at 95°C in Laemmli sample buffer (Bio-Rad) supplemented with 5% ß-mercaptoethanol added extemporaneously, resolved by electrophoresis on a 10% polyacrylamide SDS-PAGE gel and electroblotted onto Millipore Immobilon-P membranes (Millipore) in transfer buffer (25 mM Tris- HCl pH 8.0, 192 mM glycine, 20% methanol and 0.1% SDS). Membranes were blocked for one hour at room temperature in 5% blotto in TBST pH 7.5 (10 mM Tris- HCl, 0.15 mM NaCl and 0.05% Tween 20), then incubated overnight at 4°C with primary antibody. The membranes were washed once for 5 min, 10 min and 15 min in TBST, incubated for 1 h in secondary antibody at room temperature for CUGBP1 (Upstate) and for actin (gift from M. Hernandez), and washed once for 5 min, 10 min and 15 min in TBST. Antibody binding was visualized using ECLTM Western blotting analysis system and ECL plus western blotting detection system (PerkinElmer).

Western blotting was reproduced at least three times for each tissue to perform semi-quantitative analysis. Densitometric analysis of protein levels was performed using Quantity One-1-D Analysis Software (Bio-Rad Laboratories) using non-saturated exposures.

### Serum analyses

Briefly, mice were fasted overnight for 14–16 h, and blood was collected from fasted mice by retro-orbital plexus bleeding. Serum was collected by clotting blood 15 min at room temperature and centrifugation for 5 min at 10,000 g. Supernatant was taken for subsequent analyses of mouse IGF-I, GH and insulin by commercial ELISAs (Mediagnost, DSL, Mercodia, respectively) according to manufacturer's instructions. IGF binding protein 3 (IGFBP-3) levels were analyzed from serum samples by quantitative western ligand blot as described [42], [43]. Using recombinant human IGFBP-3 as internal standard on each blot, concentrations were quantitatively assessed in µg/ml.

### Immunohistochemistry

Frozen transverse serial sections of muscles (10 µm) were air-fixed for 15 min and incubated at room temperature for 1 hour in blocking solution. Sections were then washed once in PBS and incubated with four antibodies: a rabbit polyclonal antibody directed against laminin (Dako) and 3 mouse monoclonal antibodies with different isotype directed against type 1 (BA-D5, IgG2b, DSHB), 2a (SC-71, IgG1, DSHB) and 2b (BFF3, IgM, DSHB) or 2× (6H1, IgM, DSHB) MHC. Sections were then washed in PBS and incubated for 1 hour at ROOM TEMPERATURE with secondary antibodies (Alexa Fluor 488 goat anti-rabbit IgG, Cy3 anti-mouse IgG1, alexa 350 anti-mouse IgG2b, and alexa 647 anti-mouse IgGM). After several washes in PBS, slides were mounted in a mounting solution. Images were acquired with a video camera mounted on a fluorescence microscope attached to a computer. Analyses of the staining, the fibers cross sectional area (CSA_f_) and quantity were made using the Metavue software (Molecular Devices).

### Oil Red O staining

For lipid coloration, 10 µm TA transverse cryo-sections were fixed in 4% formaldehyde, 2% CaCl_2_ at 4° for 30 min and then stained with 0,5% Oil Red O for 15 min at 37°. Light microscopy was performed using an upright microscope (DMR, Leica) and a 100× objective (Leica). Images were captured using a monochrome camera (DS-Ri1, Nikon) and NIS-Elements BR software (Nikon). Twenty to thirty fields of view within the muscle section were analyzed for each animal. Images from each file were saved as grayscale images (Tagged Image File Format) and the digitized data were then analyzed with the free software Image J. Oil Red staining was quantified by establishing thresholds for the intensity of staining using the image analysis software. The lipid accumulation was calculated as total area occupied by lipid droplets of muscle fiber ×100/total cross sectional area of each field and a mean was then calculated for each subject.

### Preparation of cytosolic fractions

Tibialis anterior from DMSXL and non-transgenic mice were homogenized in an Ultra-Turrax homogenizer (low setting, 3 s) using a cytosolic extraction buffer containing: 50 mM Tris-HCl (pH 7.5), 250 mM sucrose, 5 mM MgCl_2_, 2 mM ATP, 1 mM DTT, 0.5 mM EDTA, and 0.025% digitonin, as previously reported [44]. The homogenates were centrifuged at 20,000 g for 15 min at 4°C. The pellets were discarded and the supernatants contained the cytosolic fraction [44]. Protein quantification was performed using the Bradford method (Pierce), using bovine serum albumin as a standard.

### Proteasome peptidase activities

Peptidase activities of the proteasome were evaluated using appropriate fluorogenic substrates as previously described [45]. Chymotrypsin-like, trypsin-like and caspase-like activities of the proteasome were assayed using the fluorogenic peptides LLVY-AMC (25 µM) and RLR-AMC (40 µM) and LLE-AMC (60 µM), respectively [44]. The assay buffer was composed of 50 mM Tris–HCl (pH 7.5), 40 mM KCl, 5 mM MgCl_2_, 1 mM DTT containing the appropriated peptide substrate. Enzymatic kinetics was carried out for 30 min at 37°C using 40 µg of cytosolic protein fractions from tibialis anterior in a temperature-controlled microplate fluorimetric reader (Fluostar Galaxy, bMG, Stuttgart, Germany). The excitation/emission wavelengths were 350/440 nm. The rate of proteolysis was determined for each peptide concentration by comparing the linear response of fluorescence with a standard curve of AMC. Reactions were performed in the presence (20 µM) or absence of the proteasome inhibitor N-Cbz-Leu-Leu-leucinal (MG132), to test the specificity of the measured activity.

### Measurement of contractile properties

Contractile properties of tibialis anterior and soleus muscles were evaluated by measuring the *in situ* isometric contraction in response to electric stimulation as previously described [42], [43].

### *In vivo* non-invasive dynamometric measurements

*In vivo* measurement of the ankle dorsi- and plantarflexion torque were performed with a home built dynamometer similar to the one described by Ridgley *et al.* [46]. Four-month-old WT and DMSXL female mice were anaesthetized by intra-peritoneal injection of a combination of ketamine, xylazine and midazolam (80, 15 and 0.75 mg/kg body weight, respectively). Hair on the lower hind limbs was removed and the animal was placed in supine position with hip, knee and ankle at 90°. The knee was mechanically clamped. The foot was fixed to a foot plate connected to a torque transducer so that the rotation axis for dorsi- and plantarflexion of the ankle joint was aligned with the rotation axis of the transducer. The leg length was measured in this position using a caliper. Disposable pre-sterilized electrodes (Teca, 902-DMF37) were subcutaneously inserted at the level of the tibialis anterior or of the Gastrocnemius following strict standardized operating procedure. The maximal isometric torque tetanus was elicited by direct application on the muscle of electrical stimulation with the following characteristics: 300 ms train of 200 µs biphasic square pulses at 100 Hz. This frequency was supramaximal to trigger a maximal fused tetanus in C57BL/6 mice dorsiflexors [47] and plantarflexors [48]. The optimal stimulation intensity eliciting the maximal tetanus torque was adjusted and ranged from 3 to 10 mA. The ankle torque was measured with a torque transducer (SCAIME DH 15 - 0.05 Nm) connected to a computer through a data acquisition system. A custom made LabView software program was used for data recording and analysis. Sampling frequency was set at 10 kHz. Peak torque, half-relaxation time (time for tetanic torque at the end of stimulation to decline to 50%) and late relaxation time (time for torque to decline from 50% to 10% of the tetanic torque at the end of stimulation [49] and myotonic index (ratio of the amplitude of maximal myotonic strength divided by the tetanic strength at the end of stimulation) [50] were determined. The measurements were performed in the following order to prevent ischemia by the foot plate fixation: right ankle dorsiflexion, left ankle dorsiflexion, right ankle plantarflexion and left ankle plantarflexion. For each mouse, the mean of the right and left measurements was calculated for dorsi- and plantarflexion and used for further analysis.

### Griptest

Mice were assessed for grip strength performance using a commercial grip strength dynanometer (Bioseb, Chaville, France) as previously described [22], [51].

### Wheel running test

Individually caged mice were allowed to move freely on unidirectional rotating wheels for exercise. For each cage, the running wheel was equipped with a small magnet and a bicycle speedometer (Sigma Sport BC 800) attached on the outside of the cage to measure total accumulated running distance, time spent running and speed. Every morning, the activity of the night was collected. The mice were followed over a period of 10 days.

### Treadmill test

The mice ran on a double-lane treadmill (LE8709, Bioseb, France). A shock grid that delivered 0.2 mA was placed 10 cm from the rear of the cell to provide a stimulus to make the animals run. The protocol consisted of four runs leading to exhaustion. A single trial was performed per day and each trial consisted of a run at constant speed. Four speeds were tested in each mouse. The time that the mice were able to run was recorded at each speed and was arbitrarily limited to 45 min or exhaustion. Two parameters were used to estimate endurance performance: the distance (in meters) the mice were able to cover at a given speed and the time to cover the distance (limit time, seconds). The Critical Speed (CSp) was calculated from the slope of the regression line, plotting the distance vs. the time to exhaustion from the four tests, according to the equation y = ax+b.

### Statistical analysis

All statistical analysis was performed with the StatView software (SAS Institute INC.) using two-tailed Student's t test when distributions were normal with equal or unequal variance as appropriate, Mann-Whitney test (for non-Gaussian distributions) or Chi-square test. Statistical analysis of the dynamometric measurements *in vivo* was performed with repeated measures analysis of variance (ANOVA) with population (WT vs DMSXL) as a between factor. The significance level was set at 0.05 for all statistical analyses.

## Supporting Information

Figure S1 Comparison between the mouse *Dmpk* gene and the human *DMPK* transgene expression in transgenic mice. The ratio between the mouse and human genes was studied by qRT-PCR in homozygous and hemizygous DMSXL mice (A), or by Rinonuclear Protection Assay in hemizygous DM300 mice (B). H, heart; FC, frontal cortex; B whole brain; G, gastrocnemius; K, kidney; L, liver; hem., hemizygotes; hom., homozygotes.
(TIF)

Figure S2 *DMPK* sense and antisense transcripts form nuclear foci in DMSXL heart. (A) Localization of sense and antisense *DMPK* was studied using 5′-cy3 - (CAG)5 (recognizing sense transcripts in red) and 5′-Alexa 488- (CTG)5 (recognizing antisense transcripts in green) probes in the same experiment. (B) FISH and immunohistochemistry were performed on homozygous DMSXL mouse heart using MBNL1, MBNL2 antibodies (in green) and a 5′-cy3- (CTG)5 probe recognizing *DMPK* antisense foci (in red). (C) FISH and immunohistochemistry were performed on homozygous DMSXL mouse heart using MBNL1, MBNL2 antibodies (in green) and a 5′-cy3-(CAG)5 probe recognizing *DMPK* sense foci (in red).
(TIF)

Figure S3 *DMPK* sense and antisense transcripts form nuclear foci in DMSXL skeletal muscle. (A) Localization of sense and antisense *DMPK* was studied using 5′-cy3-(CAG)5 (recognizing sense transcripts in red) and 5′-Alexa 488-(CTG)5 (recognizing antisense transcripts in green) probes in the same experiment. (B) FISH and immunohistochemistry were performed on homozygous DMSXL mouse gastrocnemius muscle using MBNL1, MBNL2 antibodies (in green) and a 5′-cy3-(CTG)5 probe recognizing *DMPK* antisense foci (in red). (C) FISH and immunohistochemistry were performed in homozygous DMSXL mouse gastrocnemius muscle using MBNL1, MBNL2 antibodies (in green) and a 5′-cy3-(CAG)5 probe recognizing *DMPK* sense foci (in red).
(TIF)

Figure S4 *DMPK* antisense and sense transcripts form nuclear foci that co-localize with MBNL1 in human DM1 heart. (A) Detection of antisense and sense *DMPK* transcripts using 5′-cy3 -(CTG)5 (recognizing antisense transcripts in red) and 5′-cy3 -(CAG)5 (recognizing sense transcripts in red) probes. (B) FISH and immunohistochemistry were performed using MBNL1 antibody (in green) and 5′-cy3-(CTG)5 or 5′-cy3-(CAG)5 probes (in red).
(TIF)

Figure S5 Splicing analysis in DMSXL mice. Alternative exon inclusions in mRNA transcripts were studied by RT-PCR in 2- and 4-month-old mice in tibialis anterior (A) and heart (B), and in various muscles from 2-month-old mice (C). Results were compared between DMSXL (n = 6) and WT (n = 6). Alternative exons studied are indicated as well as 18S (loading control). NN: mRNA from neonates.
(TIF)

Figure S6 CELF1 protein levels in WT and DMSXL mice. (A) Western blot analysis was performed on heart of 2-month-old DMSXL and WT littermate controls. (B) Proteins level were quantified by densitometric analysis using non-saturated exposures of three different membranes. Data are expressed as means ± standard deviation.
(TIF)

Figure S7 Expression of the human *DMPK* transgene in mice carrying different CTG repeat lengths. Expression of the human *DMPK* transgene was studied in heart of 2-month-old DMSXL and DM300 hemizygotes (n = 5 per group). a.u.: arbitrary units. Data are presented as means ± SEM (*p<0.05, Student's t test).
(TIF)
