# Supplementary figures and images for: Molecular, Physiological, and Motor Performance Defects in DMSXL Mice Carrying >1,000 CTG Repeats from the Human DM1 Locus
Source: PLoS Genet. 2012 Nov 29;8(11):e1003043. doi: 10.1371/journal.pgen.1003043 (PMC3510028; doi:10.1371/journal.pgen.1003043)

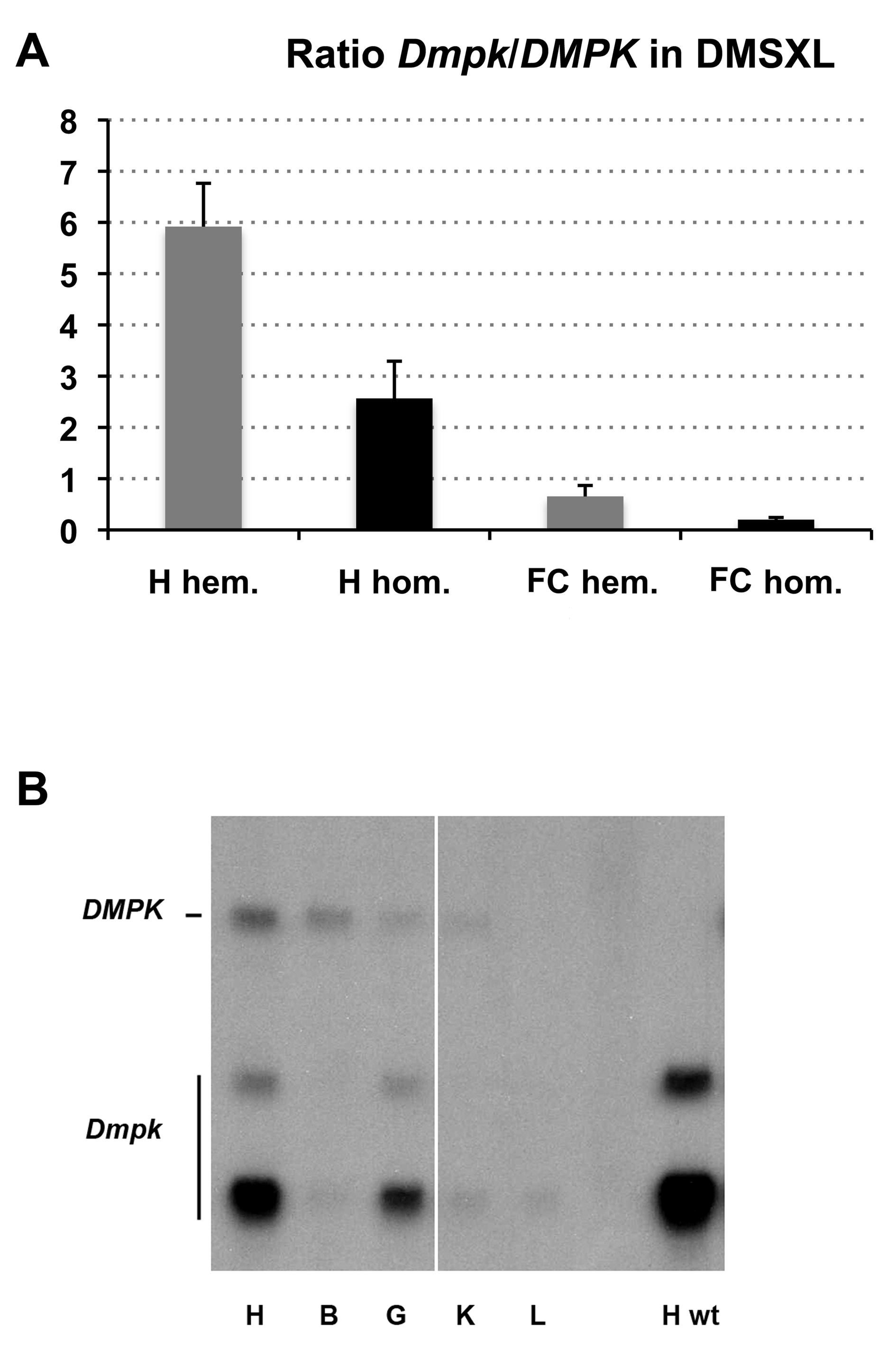

Supplement: Figure S1 — Comparison between the mouse Dmpk gene and the human DMPK transgene expression in transgenic mice. The ratio between the mouse and human genes was studied by qRT-PCR in homozygous and hemizygous DMSXL mice (A), or by Rinonuclear Protection Assay in hemizygous DM300 mice (B). H, heart; FC, frontal cortex; B whole brain; G, gastrocnemius; K, kidney; L, liver; hem., hemizygotes; hom., homozygotes. (TIF) [file pgen.1003043.s001.tif]

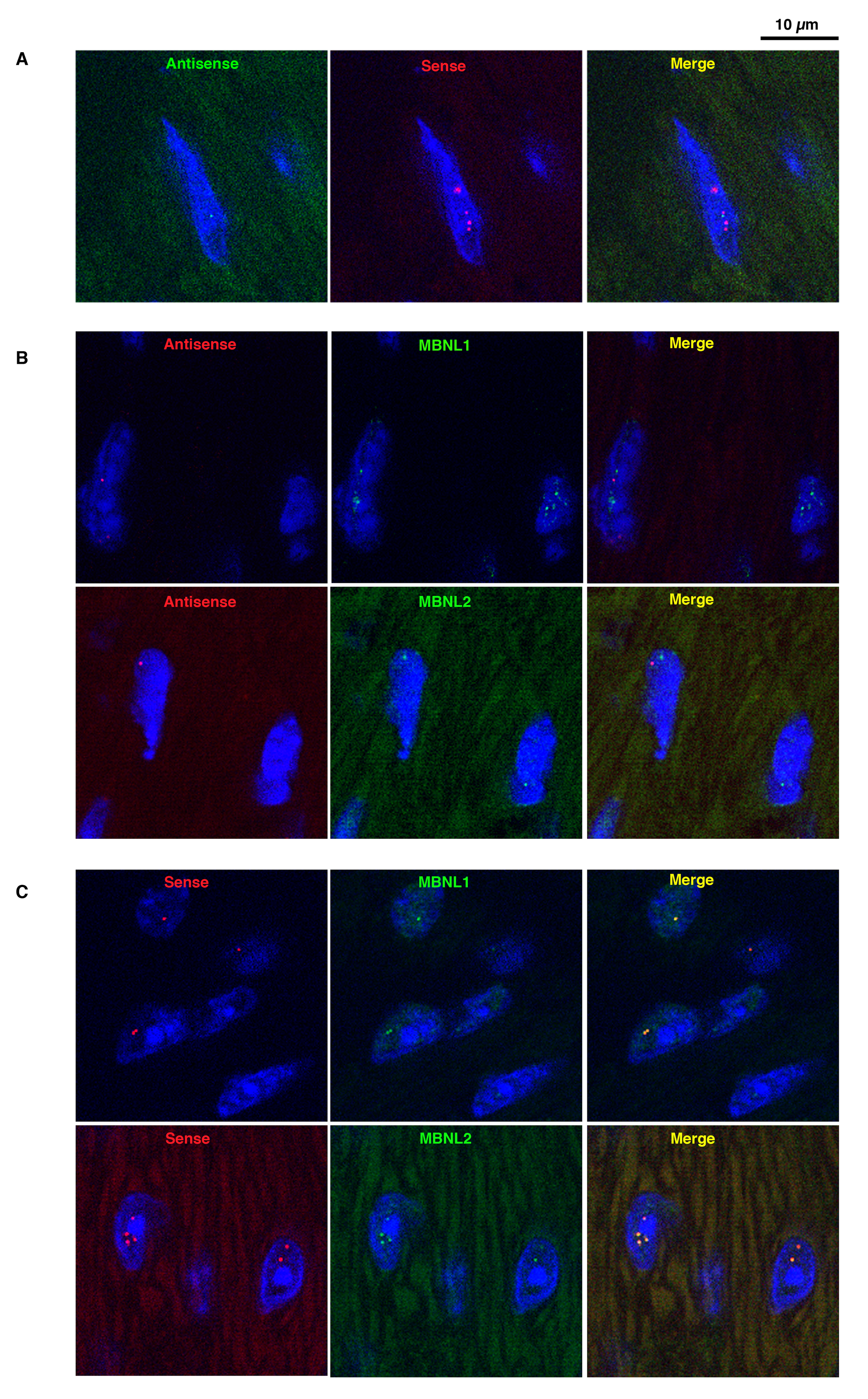

Supplement: Figure S2 — DMPK sense and antisense transcripts form nuclear foci in DMSXL heart. (A) Localization of sense and antisense DMPK was studied using 5′-cy3 - (CAG)5 (recognizing sense transcripts in red) and 5′-Alexa 488- (CTG)5 (recognizing antisense transcripts in green) probes in the same experiment. (B) FISH and immunohistochemistry were performed on homozygous DMSXL mouse heart using MBNL1, MBNL2 antibodies (in green) and a 5′-cy3- (CTG)5 probe recognizing DMPK antisense foci (in red). (C) FISH and immunohistochemistry were performed on homozygous DMSXL mouse heart using MBNL1, MBNL2 antibodies (in green) and a 5′-cy3-(CAG)5 probe recognizing DMPK sense foci (in red). (TIF) [file pgen.1003043.s002.tif]

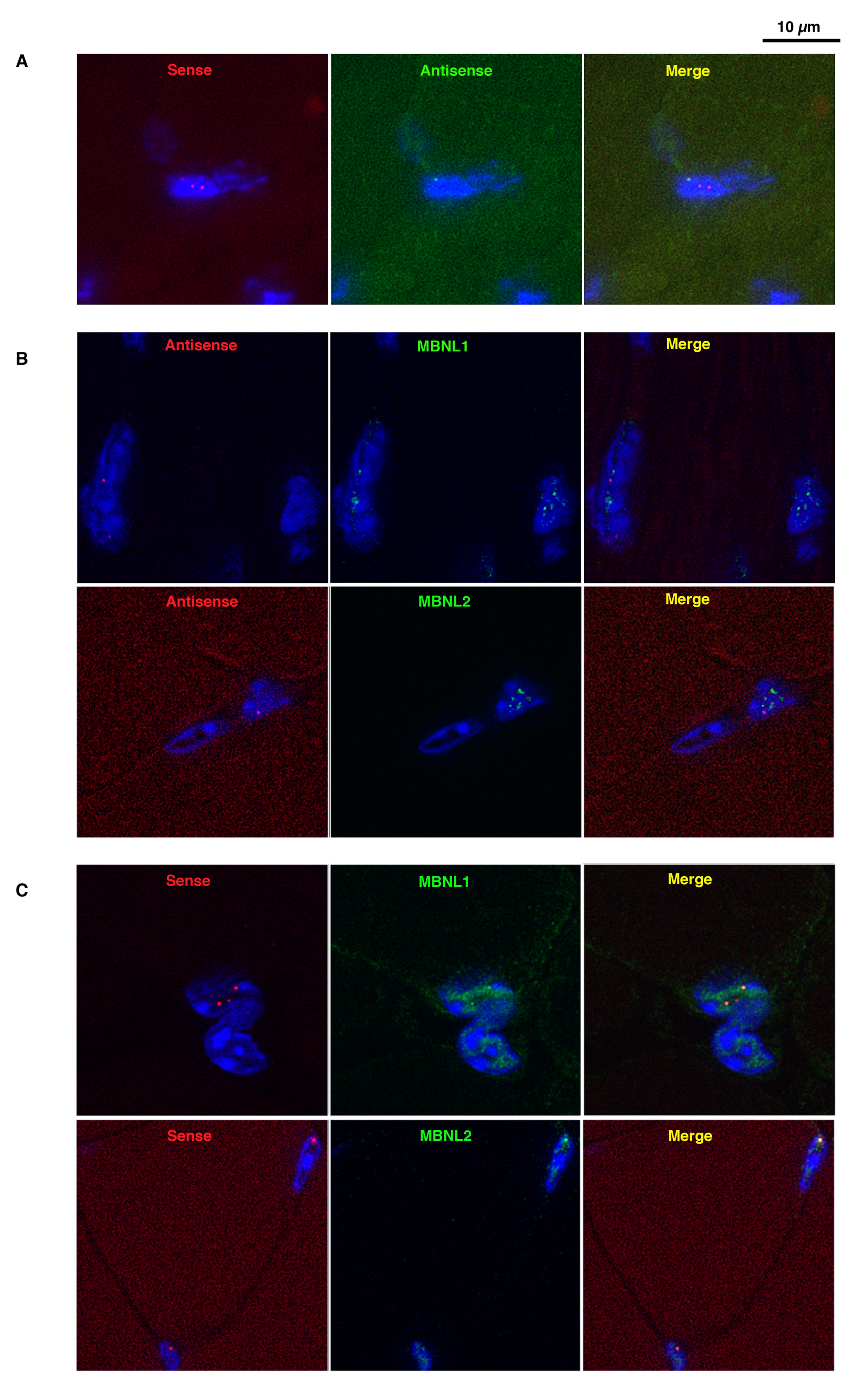

Supplement: Figure S3 — DMPK sense and antisense transcripts form nuclear foci in DMSXL skeletal muscle. (A) Localization of sense and antisense DMPK was studied using 5′-cy3-(CAG)5 (recognizing sense transcripts in red) and 5′-Alexa 488-(CTG)5 (recognizing antisense transcripts in green) probes in the same experiment. (B) FISH and immunohistochemistry were performed on homozygous DMSXL mouse gastrocnemius muscle using MBNL1, MBNL2 antibodies (in green) and a 5′-cy3-(CTG)5 probe recognizing DMPK antisense foci (in red). (C) FISH and immunohistochemistry were performed in homozygous DMSXL mouse gastrocnemius muscle using MBNL1, MBNL2 antibodies (in green) and a 5′-cy3-(CAG)5 probe recognizing DMPK sense foci (in red). (TIF) [file pgen.1003043.s003.tif]

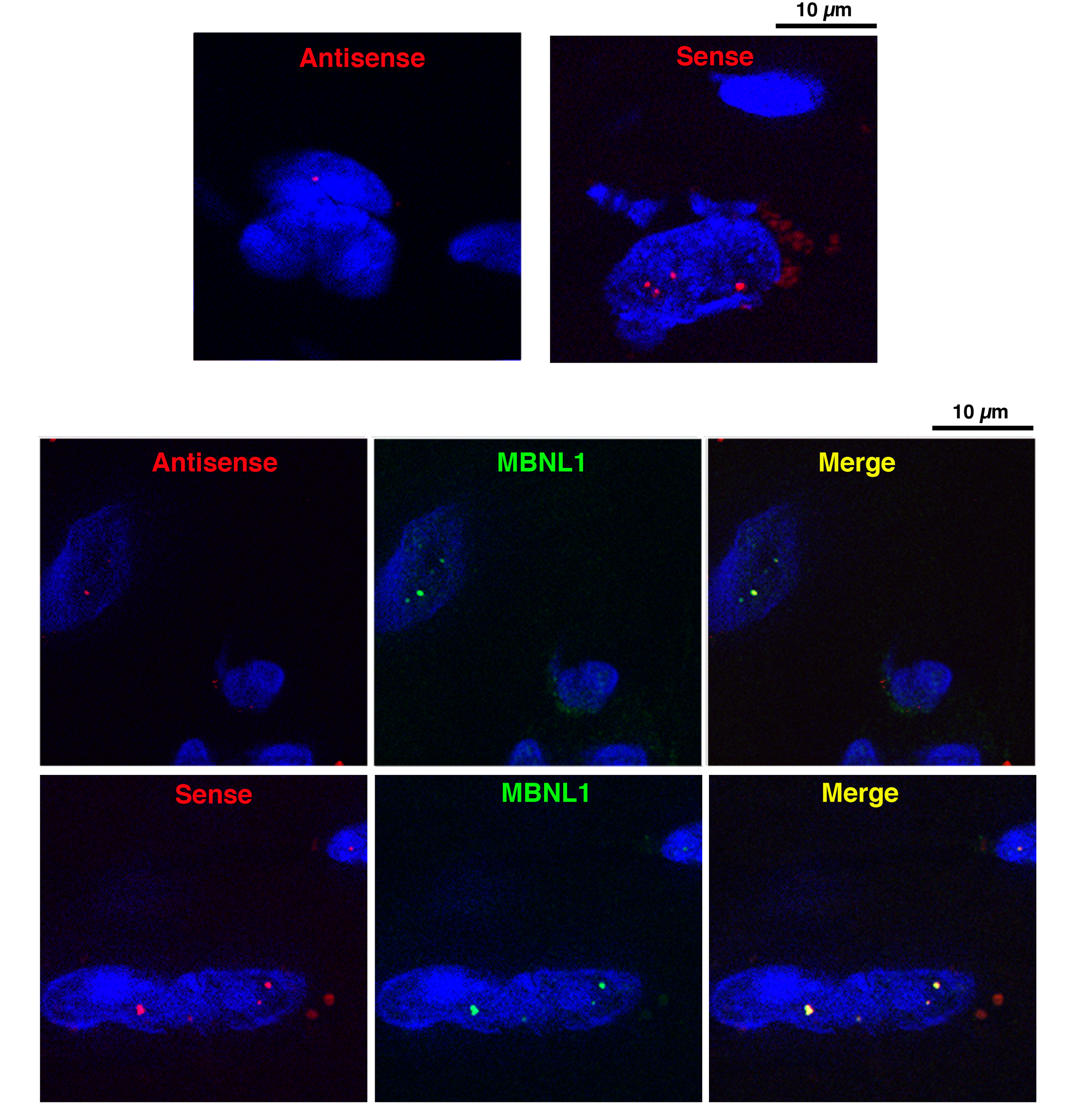

Supplement: Figure S4 — DMPK antisense and sense transcripts form nuclear foci that co-localize with MBNL1 in human DM1 heart. (A) Detection of antisense and sense DMPK transcripts using 5′-cy3 -(CTG)5 (recognizing antisense transcripts in red) and 5′-cy3 -(CAG)5 (recognizing sense transcripts in red) probes. (B) FISH and immunohistochemistry were performed using MBNL1 antibody (in green) and 5′-cy3-(CTG)5 or 5′-cy3-(CAG)5 probes (in red). (TIF) [file pgen.1003043.s004.tif]

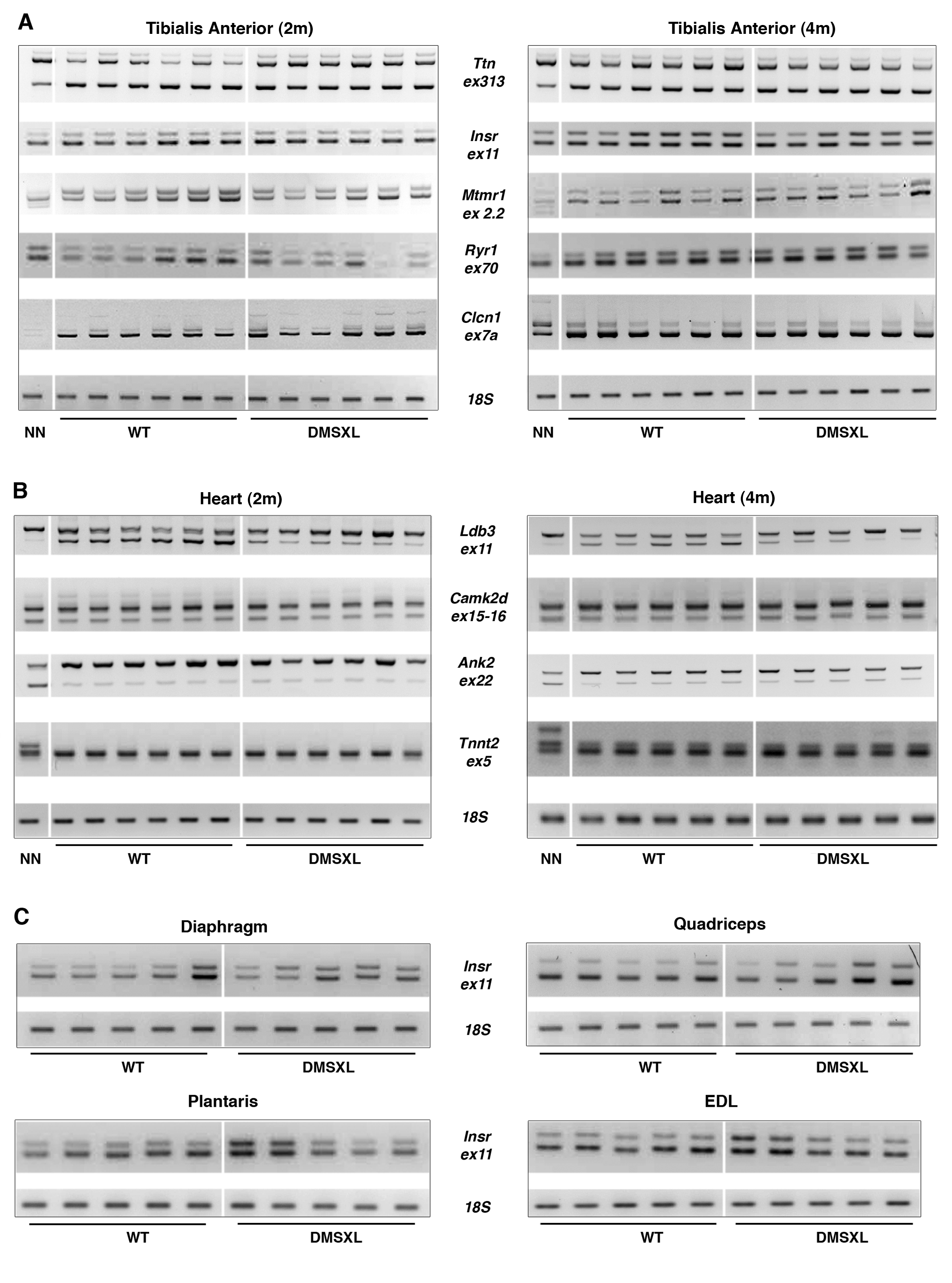

Supplement: Figure S5 — Splicing analysis in DMSXL mice. Alternative exon inclusions in mRNA transcripts were studied by RT-PCR in 2- and 4-month-old mice in tibialis anterior (A) and heart (B), and in various muscles from 2-month-old mice (C). Results were compared between DMSXL (n = 6) and WT (n = 6). Alternative exons studied are indicated as well as 18S (loading control). NN: mRNA from neonates. (TIF) [file pgen.1003043.s005.tif]

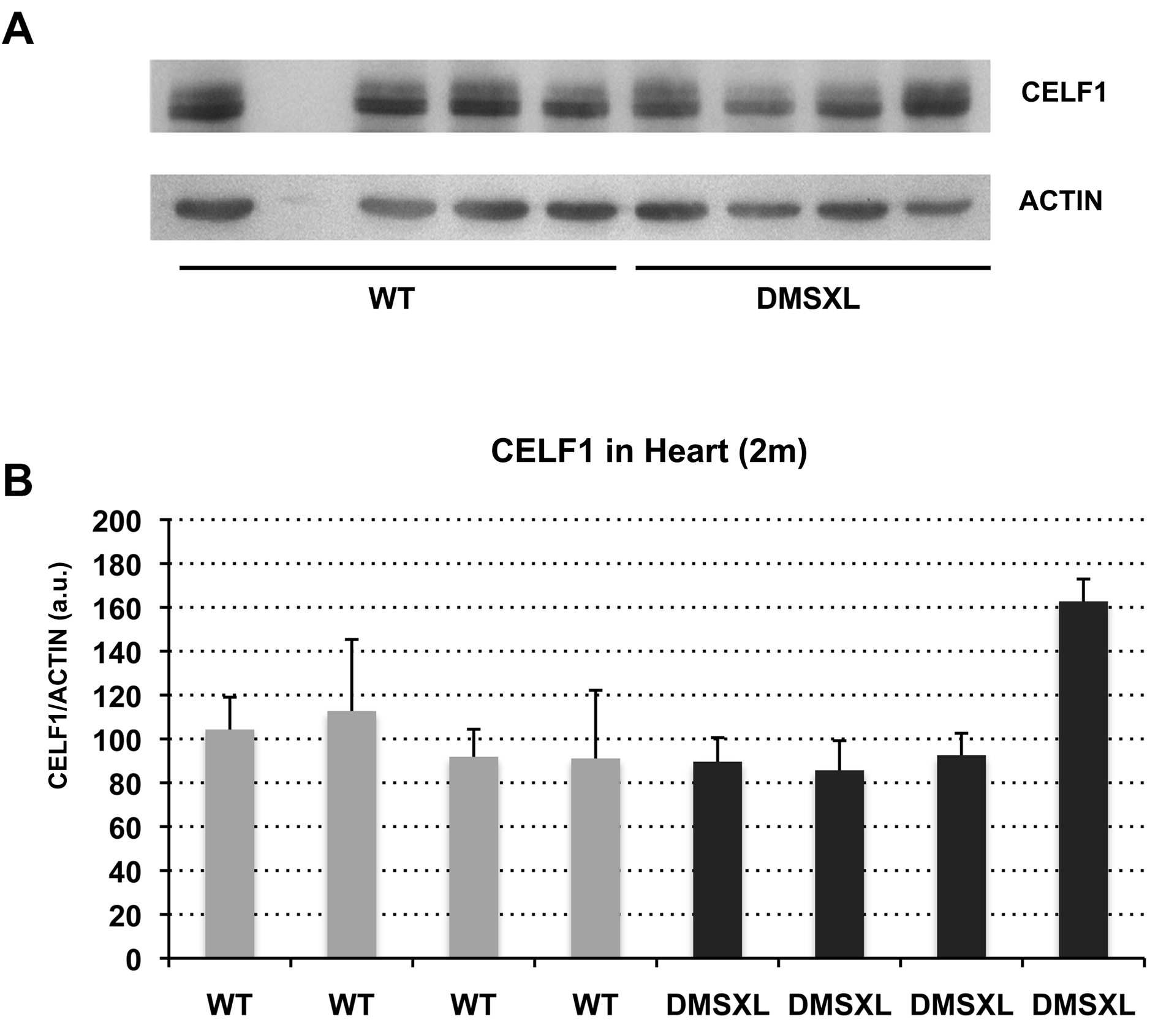

Supplement: Figure S6 — CELF1 protein levels in WT and DMSXL mice. (A) Western blot analysis was performed on heart of 2-month-old DMSXL and WT littermate controls. (B) Proteins level were quantified by densitometric analysis using non-saturated exposures of three different membranes. Data are expressed as means ± standard deviation. (TIF) [file pgen.1003043.s006.tif]

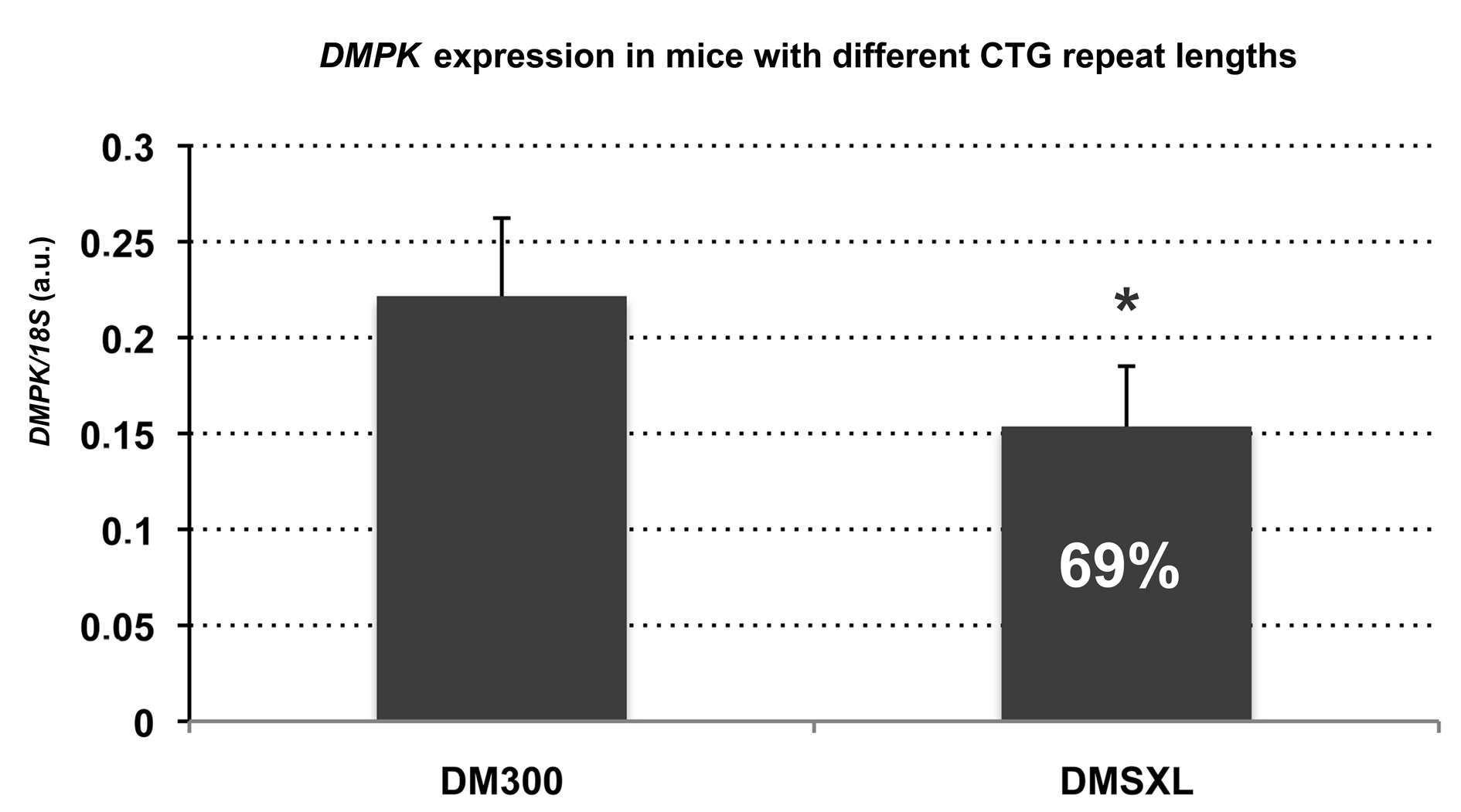

Supplement: Figure S7 — Expression of the human DMPK transgene in mice carrying different CTG repeat lengths. Expression of the human DMPK transgene was studied in heart of 2-month-old DMSXL and DM300 hemizygotes (n = 5 per group). a.u.: arbitrary units. Data are presented as means ± SEM (*p<0.05, Student's t test). (TIF) [file pgen.1003043.s007.tif]
